# MICAL1 promotes the proliferation in acute myeloid leukemia and is associated with clinical prognosis and immune infiltration

**DOI:** 10.1007/s12672-024-01150-6

**Published:** 2024-07-12

**Authors:** Yinsen Song, Zhenzhen Yang, Na Gao, Bojun Zhang

**Affiliations:** 1 Translational Medicine Research Center (Key Laboratory of Organ Transplantation of Henan Province), The Fifth Clinical Medical College of Henan University of Chinese Medicine (Zhengzhou People’s Hospital), Zhengzhou, China; 2 Department of Pathogenic Microbiology and Immunology, School of Basic Medical Sciences, Xi’an Jiaotong University, No.76 Yanta West Road, Xi’an, China

**Keywords:** Acute myeloid leukemia, Metabolism‐related genes, Tumor Immune Microenvironment, Prognostic model, MICAL1, Biomarker

## Abstract

Acute myeloid leukemia (AML) is one of the most common hematopoietic malignancies that has a poor prognosis and a high rate of relapse. Dysregulated metabolism plays an important role in AML progression. This study aimed to conduct a comprehensive analysis of MRGs using TCGA and GEO datasets and further explore the potential function of critical MRGs in AML progression. In this study, we identified 17 survival-related differentially expressed MRGs in AML using TCGA and GEO datasets. The 150 AML samples were divided into three molecular subtypes using 17 MRGs, and we found that three molecular subtypes exhibited a different association with ferroptosis, cuproptosis and m6A related genes. Moreover, a prognostic signature that comprised nine MRGs and had good predictive capacity was established by LASSO-Cox stepwise regression analysis. Among the 17 MRGs, our attention focused on MICAL1 which was highly expressed in many types of tumors, including AML and its overexpression was also confirmed in several AML cell lines. We also found that the expression of MICAL1 was associated with several immune cells. Moreover, functional experiments revealed that knockdown of MICAL1 distinctly suppressed the proliferation of AML cells. Overall, this study not only contributes to a deeper understanding of the molecular mechanisms underlying AML but also provides potential targets and prognostic markers for AML treatment. These findings offer robust support for further research into therapeutic strategies and mechanisms related to AML, with the potential to improve the prognosis and quality of life for AML patients. Nevertheless, further research is needed to validate these findings and explore more in-depth molecular mechanisms.

**Supplementary Information:**

The online version contains supplementary material available at 10.1007/s12672-024-01150-6.

## Introduction

Acute myeloid leukemia (AML) is a cancer that threatens human health by causing malignant clonal proliferation as a result of genetic abnormalities [1]. AML occurs worldwide, but the incidence rates may vary in different regions. Generally, AML is more common in European and North American countries compared to Asian and African regions. Globally, the annual rate of new AML diagnoses is approximately 4 to 5 cases per 100,000 people [2, 3]. The incidence of AML rises with age, especially among individuals aged 60 and above, where it is most prevalent. While children and young adults can also develop AML, it is relatively less frequent in this younger age cohort [4]. Some radiation and chemical exposures, such as high doses of ionizing radiation or certain chemicals like benzene and benzopyrene, are associated with an increased risk of AML [5]. These exposures are typically occupational or environmental in nature. People who have undergone radiation therapy or chemotherapy for other cancers may be more susceptible to developing AML because these treatments can damage normal blood-forming cells [6, 7]. The primary approach for managing AML patients currently involves chemotherapy. However, despite achieving initial remission, a significant percentage of patients experience disease recurrence or succumb to the illness. Despite substantial research efforts aimed at developing targeted and combination therapies, the 5 year survival rate for AML patients remains below 30%. Consequently, it is imperative to discern innovative prognostic biomarkers for monitoring patient prognoses and gaining deeper insights into the pathogenesis of AML.

Dysregulated metabolism is a condition where various biochemical reactions and metabolic processes within the body are abnormally regulated. It has been shown to have a strong correlation with the development and occurrence of tumors [8]. Tumor cells typically exhibit a range of abnormal metabolic features that play pivotal roles in tumor biology [9, 10]. Firstly, tumor cells often exhibit the phenomenon known as the “Warburg effect,” wherein they generate ATP energy through lactic acid fermentation instead of the typical oxidative phosphorylation process, particularly under conditions of low oxygen. This metabolic adaptation enables tumor cells to thrive and sustain rapid growth. Additionally, tumor cells display a heightened dependency on glucose, which results in abnormal activation of glucose metabolism pathways such as glycolysis and gluconeogenesis. These adaptations are crucial for supporting the accelerated proliferation characteristic of cancer cells. Dysregulated lipid metabolism is also common, resulting in the accumulation of abnormal lipid compositions that provide essential building blocks for cell membranes and promote tumor growth. Furthermore, disruptions in nitrogen metabolism and alterations in acid–base balance are also associated with tumor development. These metabolic abnormalities can contribute to tumor initiation and progression by providing the necessary energy and materials for survival, resistance to cell death, and alteration of the extracellular microenvironment, among other mechanisms [11–13]. Certain metabolic abnormalities may increase an individual's risk of developing leukemia. For example, metabolic disorders associated with obesity have been linked to an increased incidence of certain leukemia subtypes. Some leukemia subtypes, such as Acute Lymphoblastic Leukemia (ALL) and AML, may be associated with specific metabolic abnormalities or gene mutations. These abnormalities may play a role in cell growth and proliferation [14, 15]. Leukemia treatments themselves can impact a patient's metabolism. For instance, radiation therapy and chemotherapy can induce metabolic disruptions, including anemia, nausea, loss of appetite, and other side effects, all of which can impact a patient's nutritional status and metabolic health [16, 17]. Therefore, gaining a deep understanding of and intervening in metabolic abnormalities are of paramount importance in AML treatment and research, with the potential to offer critical support for the development of more effective therapies.

The Tumor Immune Microenvironment (TME) refers to the complex network of cells and molecules surrounding tumor cells, including immune cells, blood vessels, stromal cells, cytokines, and signaling pathways [18, 19]. This environment plays a crucial role in the growth, development, and treatment response of tumors. Within the TME, various types of immune cells, including macrophages, natural killer cells, B cells, T cells, and dendritic cells, are present and play crucial roles in surveilling and combating the growth and spread of tumor cells [20, 21]. However, tumor cells can also employ various strategies to evade attacks from the immune system, including reducing the expression of tumor antigens and suppressing the activity of immune cells. Additionally, the TME includes the tumor-associated vascular system, which supplies nutrients and oxygen required by tumor cells, although tumor blood vessels may be abnormal, leading to inadequate oxygen supply. Understanding the TME is crucial for the development of immunotherapies designed to alleviate immune suppression within the TME and enhance the immune system's ability to target tumors [22–24]. Lastly, the characteristics of the TME can significantly influence a patient's prognosis and their response to treatment. Therefore, a comprehensive understanding of its features and dynamic changes is crucial for developing more effective cancer treatment strategies. Several studies have indicated that the immune microenvironment of AML is often abundant in immune-suppressive factors such as anti-inflammatory cytokines and immune-inhibitory cells. These factors can suppress the activity of immune cells, making it challenging for them to effectively attack AML cells. In addition, despite the presence of immune cells in the TME of AML patients, AML cells can employ various strategies to evade attacks from the immune system. These strategies include reducing the expression of tumor antigens, altering cell surface molecules to evade immune detection, and recruiting immune-suppressive cells, among others. However, the potential mechanism involved in the effects of TME on AML progression remained largely unclear.

In this study, we carried comprehensive assays using High throughput sequencing to explore the expressions of metabolism-related genes (MRGs) and their clinical significance in AML patients. Then, we analyzed the association between MRGs and TME in AML. Finally, we identified a critical metabolism-related gene MICAL1 and performed functional experiments to explore its potential function in AML. This study is a multi-faceted research effort aimed at gaining a comprehensive understanding of the expression and clinical significance of MRGs in AML patients, as well as their interactions with the TME. By identifying and investigating key genes such as MICAL1, our goal is to uncover the underlying pathological mechanisms of AML and provide novel therapeutic targets and strategies for patient treatment.

## Material and methods

### Cell culture and cell transfection

THP-1 and NB-4 cells were purchased from Shanghai Bioleaf Biotech company (Shanghai, China). The cells were routinely cultured in phenol red-positive RPMI 1640 media supplemented with 10% (v/v) fetal bovine serum. The small interfering RNAs (siRNAs) targeting MICAL1 (si-MICAL1-1, si-MICAL1-2) were synthesized by Shanghai Generay Technologies (Pudong, Shanghai, China). The Lipofectamine^™^ 3000 was employed to transfect siRNAs into TPH-1 and NB-4 cells. Briefly, 10 μl Lipofectamine^™^ 3000 reagent was diluted with 490 μl OpitiMEM (Gibco, Carlsbad, CA, USA), and si-MICAL1-1, si-MICAL1-2 or si-Control (each 5 μl) was also was diluted with 490 μl OpitiMEM. After combining the LipofectamineTM 3000 and siRNA solutions and letting them incubate for 5 min, we poured 500 μl of the resulting mixture into a single well of 6-well plates. The cells were prepared for studies 48 h after transfection, at which time the medium was replaced (at 6 h post-transfection).

### Cell proliferation detection

Using a Cell Counting Kit-8 (CCK-8) (Vazyme, Nanjing, Jiangsu, China), we were able to measure the cell proliferation rates of THP-1 and NB-4 cells that had been transfected with siRNAs targeting MICAL1. Briefly, at a density of 5000 cells per well, either control cells or cells that had been transfected with siRNA were seeded onto plates that included 96 wells. After that, 10 μl of CCK-8 reagent was given to each well at four separate time intervals (24 h, 48 h, 72 h, and 96 h), and the cells were cultured at 37 degrees Celsius for two to three hours. The light absorbance at 450 nm was determined by employing a microplate reader manufactured by BioTek Instruments (Winooski, Vermont, United States).

### Data acquisition and differentially expressed genes (DEGs) obtainment

Using the TCGA database, we gathered information on the genetic mutations, transcriptomes, and clinical presentations of AML. Possemato's study yielded a total of 2752 metabolism-related genes (MRGs), which are said to code for all of the known human metabolic enzymes and transporters. We downloaded the over-expressed and down-expressed in AML tumor samples using GEPIA database. Since there were no paired normal sample data of AML in TCGA database, the GEPIA database used GTEx data, and there were 173 AML tumor samples from TCGA database and 70 normal tissue samples from GTEx database, and the total DEGs in AML (named TCGA-AML-DEGs) were 7964 (|logFC|> 1, p < 0.05). In addition, the GSE114868 data were downloaded from Gene Expression Omnibus (https://www.ncbi.nlm.nih.gov/geo/), and 1273 up-regulated and 1037 down-regulated genes (|logFC|> 1, p < 0.05) were identified using R software “limma” package, and the package “ggplot2” was utilized to plot the volcano map and heatmap. Besides, 2618 genes with significant overall survival in AML from UALCAN database (UALCAN-OS) were obtained. The 17 overlap MRGs were identified using Venny 2.1.

### Functional enrichment analysis

GO and KEGG are essential bioinformatics resource designed to assist researchers in understanding the functionality of genes and genomes, as well as their roles in biological processes [25]. They are developed and maintained by the Bioinformatics Center at Kyoto University in Japan and has become a crucial tool in the field of life sciences. ClusterProfiler is an R package used in bioinformatics and biostatistics for functional enrichment analysis and visualization. Its purpose is to help researchers understand gene sets within high-throughput biological data, enabling the discovery of functional and pathway enrichment patterns associated with these gene sets in biological processes [26]. R’s “clusterprofiler” and “enrichplot” packages were used to conduct functional enrichment analysis, which included GO and KEGG pathway enrichment analyses. A false discovery rate (FDR) of 0.05 or lower was required for a functional category to be considered statistically significant.

### Construction of MRGs related prognostic signature for AML

LASSO is a statistical method used for linear regression and feature selection. It is employed in regression analysis to address high-dimensional datasets and tackle issues like multicollinearity (high correlation among multiple independent variables) and feature selection. Using the 17 overlapping MRGs, a prognostic classifier was developed using the LASSO Cox regression model. In order to do Lasso Cox regression, the “survival” and “glmnet” R packages were utilized. Besides, Multivariate cox regression analysis was another method that was applied for the purpose of obtaining the prognostic model for AML based on the 17 overlap MRGs. The Kaplan–Meier method and the “survival” package in R were utilized to compare the overall survival rates of patients categorized into high-risk and low-risk groups. Additionally, receiver operating characteristic (ROC) analysis and calculation of the area under the curve (AUC) were performed using the “survival ROC” package in R.

### Real-time PCR detection

The RNA purification kits (Invitrogen, Grand Island, NY, USA) were applied to extract the total RNAs from THP-1 and NB-4 cells transfected with siRNAs targeting MICAL1. Then SuperScript III Reverse Transcriptase kits (Thermo Fisher Scientific, Inc., Waltham, MA, USA) were employed to synthesize the complementary DNAs (cDNAs). The amplification of cDNAs were conducted with Light Cycler 480 SYBR Green I Master Mix using the 7900HT Fast Real-Time PCR System. Gene expression was standardized against GAPDH, and the 2'-Ct technique was used to assess the relative expression levels of MICAL1. The cycling conditions were as follows: 95 ℃ for 5 min, 40 cycles of 95 ℃ for 15 s, 56 ℃ for 30 s and 72 ℃ for 30 s, and 95 ℃ for 60 s. The primers were listed as follows: GAPDH: forward 5′-TCAAGAAGGTGGTGAAGCAGG-3′ and reverse 5′-TCAAAGGTGGAGGAGTGGGT-3′; MICAL1: forward 5′-TGTTGGCTGAGCGTGAGAG-3′ and reverse 5′-ATCTGTCTTGTCGTTGTTCCTC-3′.

### Molecular subtypes of AML identification

The potential molecular subtypes of AML based on these 17 overlap MRGs were investigated by using the R package “ConsensusClusterPlus”. ConsensusClusterPlus is a bioinformatics tool designed for cluster analysis. Its primary purpose is to assist researchers in identifying and evaluating the stability of potential clustering structures within high-dimensional biological data. It is typically used to analyze high-dimensional data in fields such as gene expression, protein interactions, genomics, and others, aiming to uncover hidden biological patterns within the data.The CDF and consensus matrices were used to determine the best method for classifying molecules into groups. The "ConsensusClusterPlus" R package was used to do the classification of the AML samples using the k-means algorithm, with k ranging from 2 to 6. Cluster analysis using CDF and area under the CDF curve at varying cluster sizes indicated that k = 3 was optimal for distinguishing between three distinct molecular subtypes in the data set.

### The immune related analyses

Immune cells and their distribution were analyzed across three AML molecular subtypes using the CIBERSORT algorithm. The potential response to immune checkpoint blockade (ICB) in these subtypes (G1 to G3) was assessed using the TIDE algorithm. Furthermore, immune networks involving DEGs or MICAL1 with various immune cell types in the AML subtypes (G1 to G3) were analyzed using the R software packages “immuneeconv” and visualized using “ggClusterNet”.

### Online websites for bioinformatics analyses

Gene Set Variation Analysis (GSVA) is a computational method commonly used in bioinformatics and genomics for the analysis of gene expression data. It is designed to evaluate the activity or enrichment of predefined gene sets or biological pathways within a given set of gene expression profiles. GSVA does not require a priori sample grouping or class labels and can be particularly useful for exploring functional differences in high-dimensional gene expression data. The DEGs or MICAL1 expression, various survivals, genetic changes (SNV and CNV), methylation, GSVA across pan-cancers were analyzed by using the GSCA database. The expression of MICAL1 in kinds of cancer types and their corresponding paired normal specimens were explored by the use of TIMER 2.0 database (http://timer.cistrome.org/). The MICAL1 expression in kinds of cell types from different human tissues was using Harmonizome database (https://maayanlab.cloud/Harmonizome/). GeneMania (https://genemania.org/) database to analyze genes or proteins which were able to interact or co-express with MICAL1. Using the TISIDB database, an investigation of the connections between the expression of MICAL1 and the number of tumor-infiltrating lymphocytes (TILs), immunoinhibitors, and chemokines in pan-cancers was carried out. The GEPIA database was utilized for analyzing gene expression and survivals.

### Statistical analysis

The Student's t-test was utilized in order to carry out statistical comparisons between the two different groups. When it was essential to make comparisons between more than two groups, a one-way analysis of variance (ANOVA) was used, and then post-hoc Tukey’s honest significant difference (HSD) testing was performed. Survival analyses were executed using the log-rank test. The statistical software SPSS was utilized throughout each and every one of the analyses. A significance threshold of p < 0.05 was adopted.

## Results

### The obtainment of overlap metabolism-related genes (MRGs) in AML

**Fig. 1:**
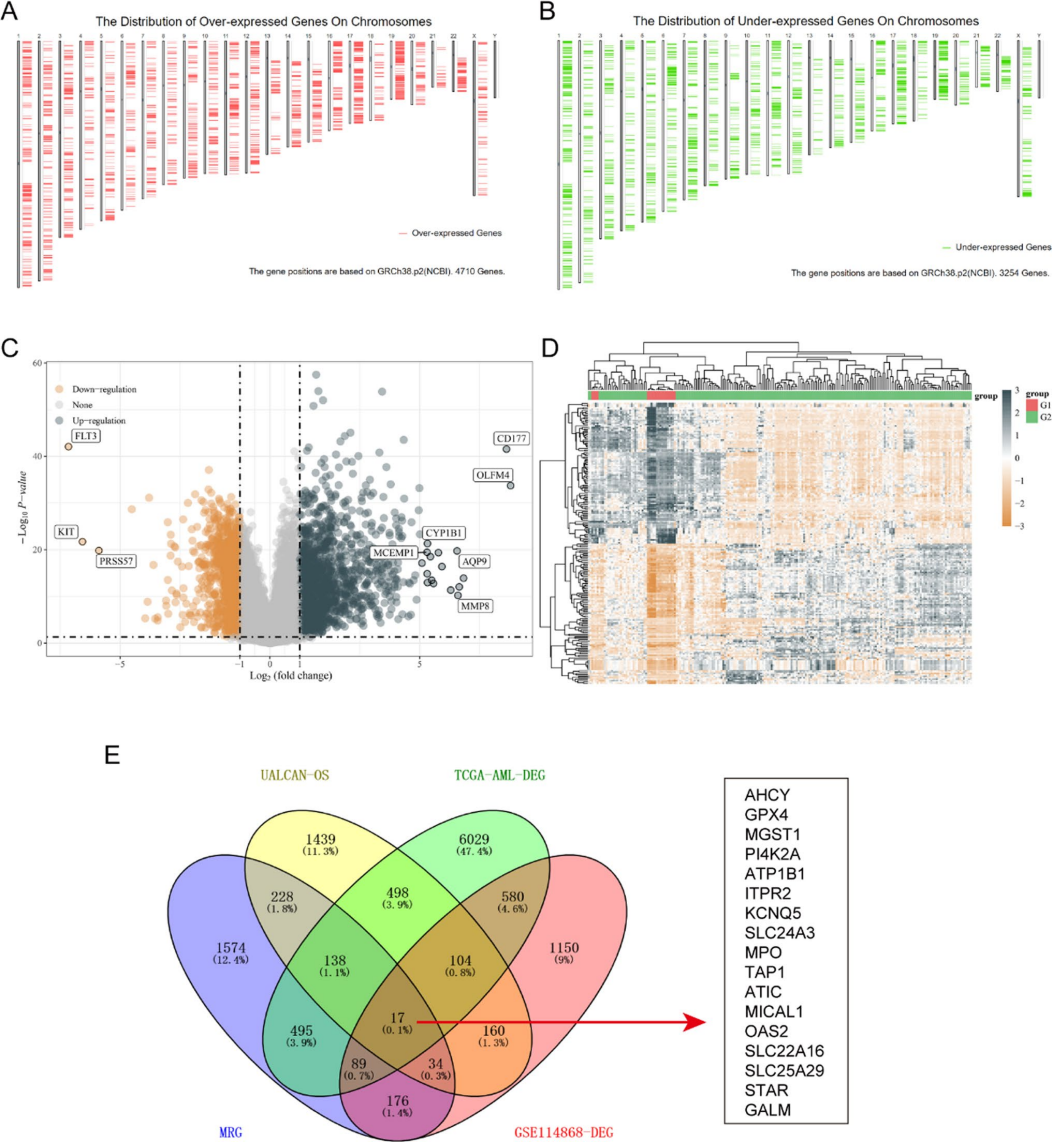
Overlap MRGs obtainment. **A** and **B** The distribution of over- and down-expressed genes on chromosomes were respectively presented. **C** Volcano plot of DEGs in GSE114868. **D** DEGs’ heatmap in GSE114868. **E** The overlap genes of MRGs, TCGA-AML-DEGs, GSE114868-DEGs and UALCAN-OS

To discover the potential genes which might be selected as possible therapy targets in AML, we first downloaded the over-expressed and down-expressed in AML tumor samples using GEPIA database. Since there were no paired normal sample data of AML in TCGA database, the GEPIA database used GTEx data, and there were 173 AML tumor samples from TCGA datasets and 70 normal samples from GTEx datasets. The distribution of over- and down-expressed genes on chromosomes were respectively presented in Fig. 1A and B, and the total DEGs in AML (named TCGA-AML-DEGs) were 7964 (|logFC|> 1). Afterwards, we analyzed the GSE114868 data to further verify the deferentially genes (DEGs) in AML, and 1273 up-regulated and 1037 down-regulated genes were obtained. The corresponding volcano plot and heatmap of these DEGs from GSE114868 data were displayed respectively in Fig. 1C and D. Then, we sought to obtain the overlap genes of MRGs (2752 genes), TCGA-AML-DEGs (7964 genes), GSE114868-DEGs (2310 genes) and UALCAN-OS (2618 genes with significant overall survival in AML from UALCAN database) using venny website, and 17 overlap MRGs were identified (Fig. 1E).

### The expression and survival analyses of 17 overlap MRGs across *cancer* types

**Fig. 2:**
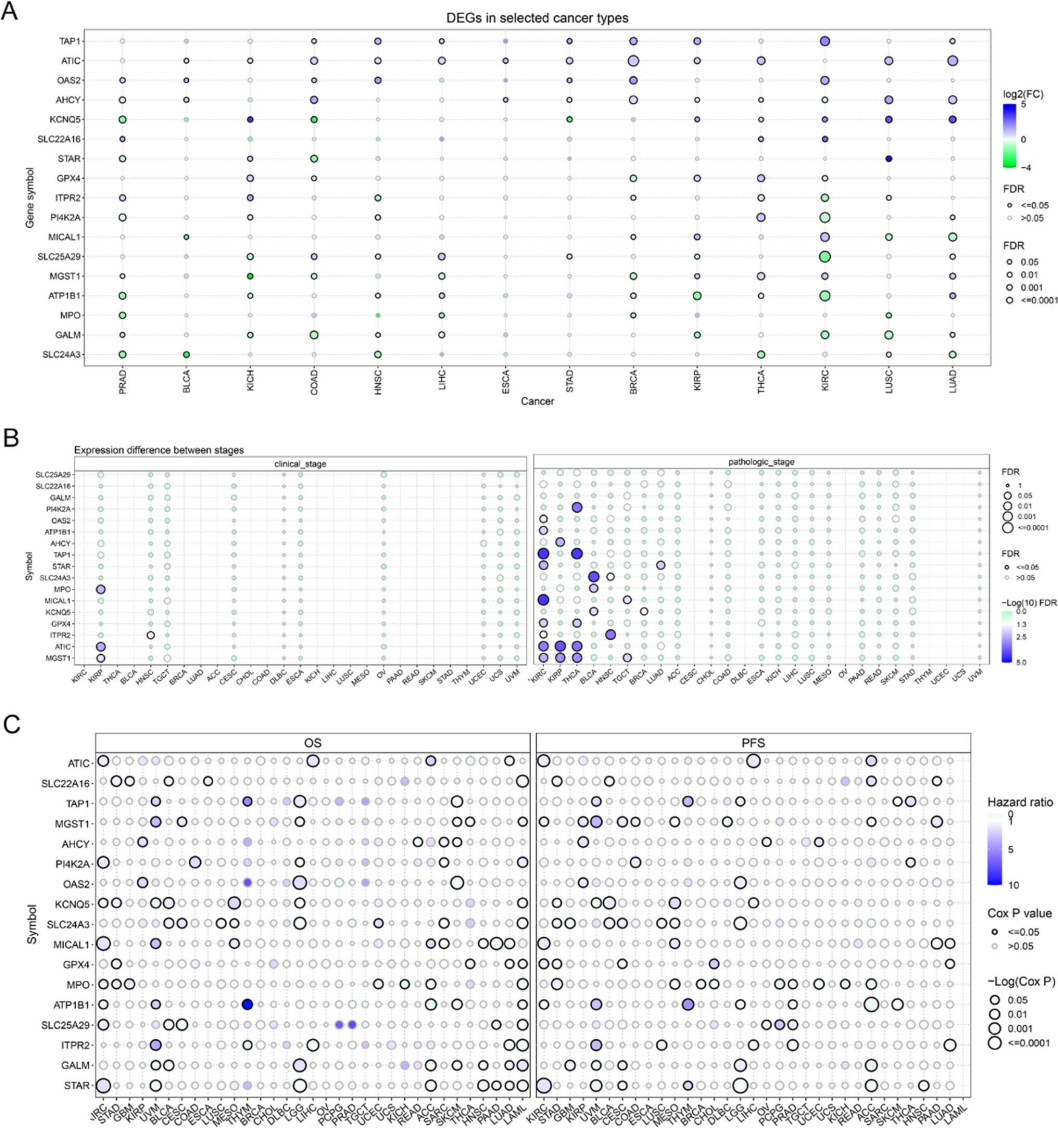
Expression and survival analysis of 17 overlap MRGs in cancers. **A** The relative mRNA levels of 17 overlap MRGs in pan-cancers. **B** The expression of the 17 overlap MRGs in clinical stages and pathological stages. **C** The analysis of the OS and PFS of the 17 overlap MRGs in cancers

Next, we employed GSCA database to study the 17 overlap MRGs’ expressions in pan-cancers. We observed that the 17 overlap MRGs exhibited different expression levels in different cancers (Fig. 2A). For instance, genes like SLC24A3, GALM, MPO, and ATP1B1 appear to be commonly down-regulated across various solid cancers, while other MRGs are frequently up-regulated in many cancer types. The expression patterns of these 17 overlapping MRGs were further investigated in relation to clinical and pathological stages. The data demonstrated that nearly all the genes had no significant difference between different clinical stages in pan-cancers, and half of the 17 overlap MRGs had obvious expression difference in pathological stages in KIRC, KIRP, THCA and BLCA but no significant difference in other cancers’ pathological stages (Fig. 2B). Moreover, the OS and PFS of the 17 overlap MRGs in cancers were assessed, and the data revealed that most of the 17 overlap MRGs had significant OS or PFS in kinds of cancer types (Fig. 2C).

### Genetics changes and methylation analyses of 17 overlap MRGs in *pan*-cancers

**Fig. 3:**
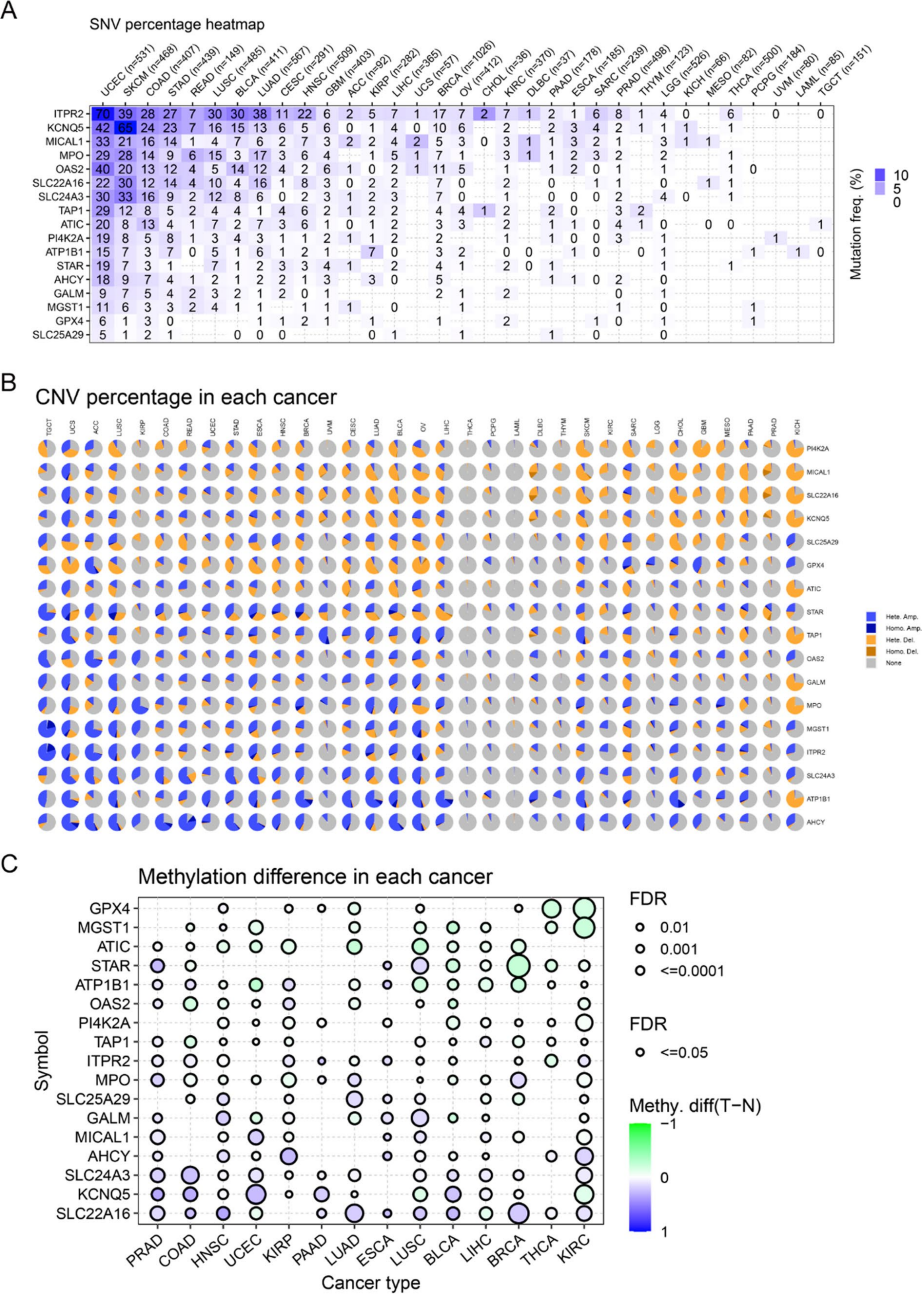
SNV, CNV and methylation analyses of overlap MRGs. **A** The SNV analysis. **B** The CNV analysis. **C** Methylation difference in each cancer types

Genetic changes including the SNV and the CNV were further evaluated. The SNV percentage heatmap from USCA database based on TCGA data elucidated that most of the 17 overlap MRGs had SNV mutation in UCEC, SKCM and COAD, while the the 17 overlap MRGs had no SNV in the majority of cancer types (Fig. 3A). Additionally, the CNV mutation frequencies of the 17 overlap MRGs were assessed, and the results of the pie plots suggested that most of 17 overlap MRGs had CNV in most cancer type except in AML, PCPG and THCA, and the CNV type of these genes was mainly heterozygous amplification (Fig. 3B). Subsequently, 17 overlap MRGs’ methylation levels were evaluated, and we found that more than half of the 17 overlap MRGs’ methylation levels in tumor samples of the majority of cancer types were higher than that in the corresponding normal tissues (Fig. 3C).

### GSVA analysis of the 17 overlap MRGs

**Fig. 4:**
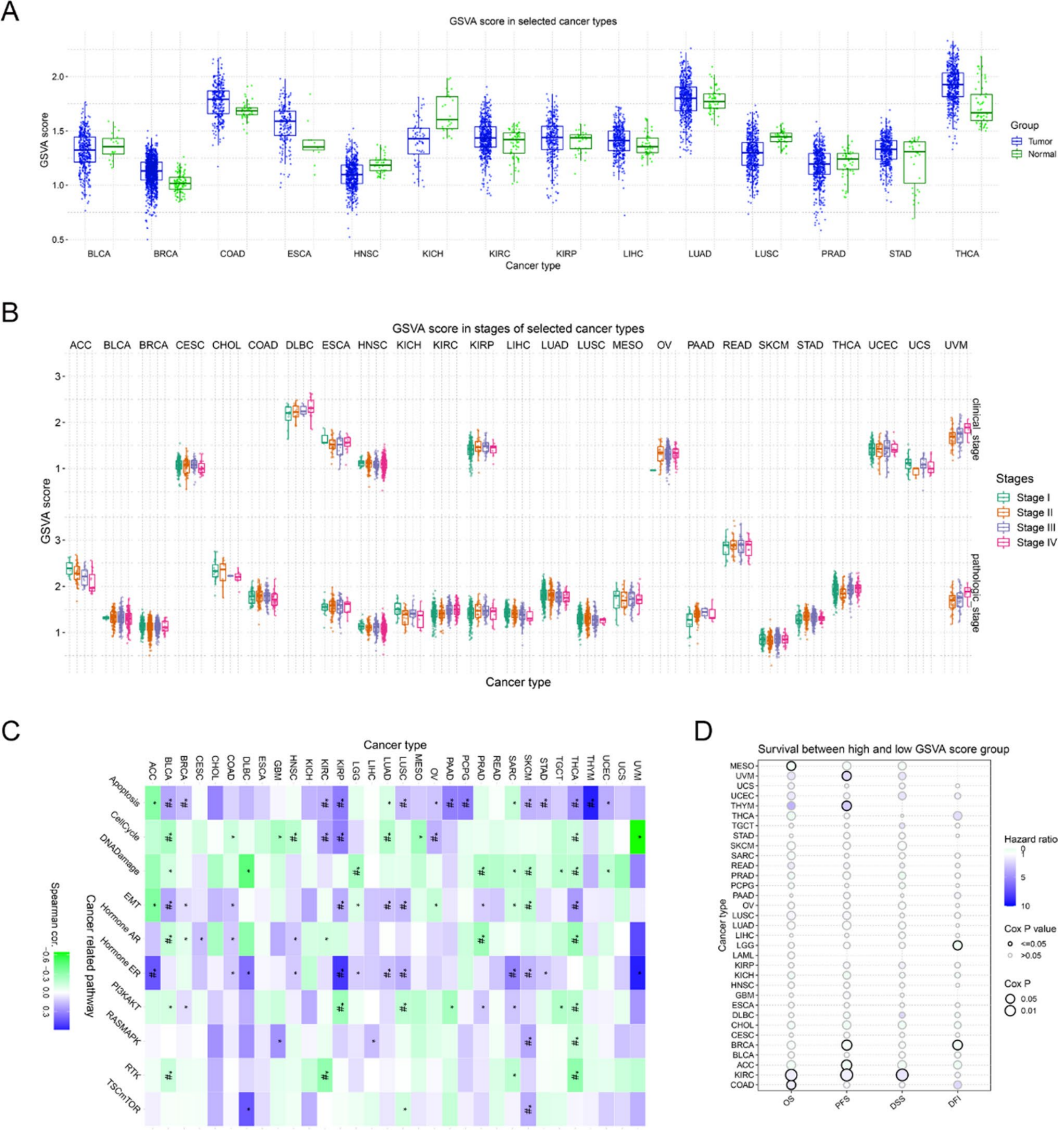
Overlap MRGs’ GSVA (Gene Set Enrichment Analysis) analysis. **A** GSVA scores of the 17 overlap MRGs in pan-cancers. **B** The analysis of the GSVA scores in clinical stages and pathological stages of pan-cancers. **C** The correlations between the GSVA scores and many signaling pathway activities. **D** The survival analyses of the high and low GSVA score groups in pan-cancers

The 17 overlap MRGs as a whole were then subjected for GSVA analysis. As the data presented in Fig. 4A, the GSVA scores of the 17 overlap MRGs were higher in tumor samples of the most cancer types than that in the corresponding normal tissues. Then, the GSVA scores in clinical stages and pathological stages of kinds of cancer types were also estimated (Fig. 4B). Furthermore, the correlations between the GSVA scores and many signaling pathway activity was also determined, and the results proved that the GSVA score consisted with 17 overlap MRGs was positive correlation with apoptosis, EMT, hormone ER, and negative correlation with DNA damage and cell cycle in many tumors (Fig. 4C). Finally, the survival analyses of the high and low GSVA score suggested that higher GSVA score had poor OS, PFS, and DSS in COAD and KIRC, and there seemed to be no significant difference in other cancer types (Fig. 4D).

### Identification of metabolism-related genes molecular subtypes of AML

**Fig. 5:**
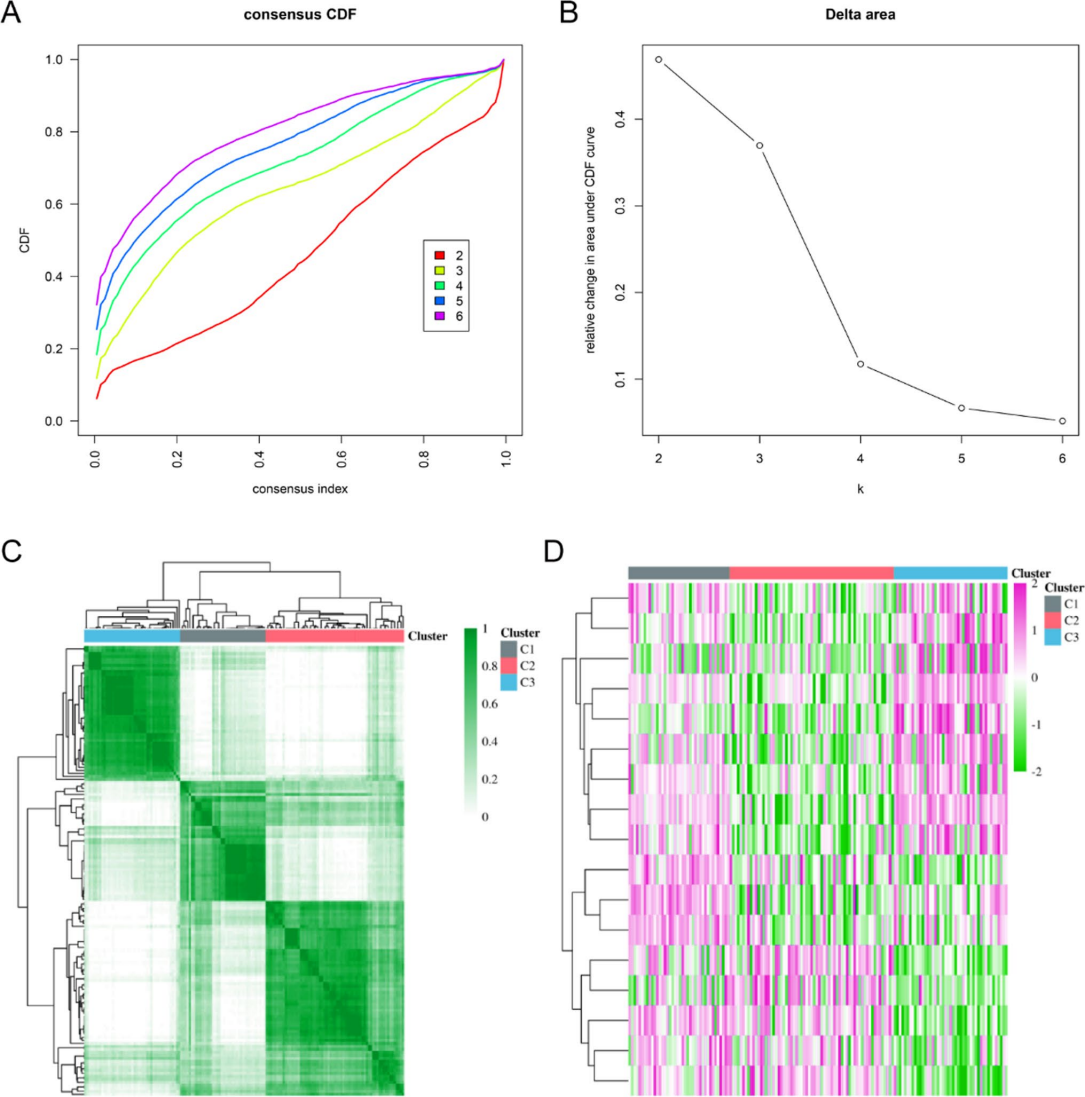
Identifcation of three AML molecular subtypes based on 17 overlap MRGs. **A** CDF from a consensus clustering sample with k between 2 and 6 subtypes. **B** Difference between the area under the k = 2 and k = 6 CDF curves. **C** Heatmap of sample clustering under k = 3. **D** the heatmap of 17 overlap MRGs in three AML molecular subtypes (C1: clustering 1; C2: clustering 2; C3: clustering 3)

We next attempted to clarify the potential molecular subtypes of AML based on these 17 overlap MRGs. CDF, or cumulative distribution function, was the method that was used to get an agreement on the best number of clusterings. Cluster number k = 3, as indicated by the area under the CDF curve in different cluster numbers, was shown to clearly classify samples into three different molecular subtype groups (Fig. 5A and B). The heatmap in Fig. 5C presented that the 150 AML tumour samples were divided into these 3 molecular subtypes including clustering 1 (C1, 40 AML samples), clustering 2 (C2, 65 AML samples), and clustering 3 (C3, 45 AML samples). Then, the relative expression of the 17 overlap MRGs in the three molecular subtypes of AML was displayed using a heatmap (Fig. 5D).

### Ferroptosis, cuproptosis and m6A related genes’ expression in the three molecular subtypes of AML

**Fig. 6:**
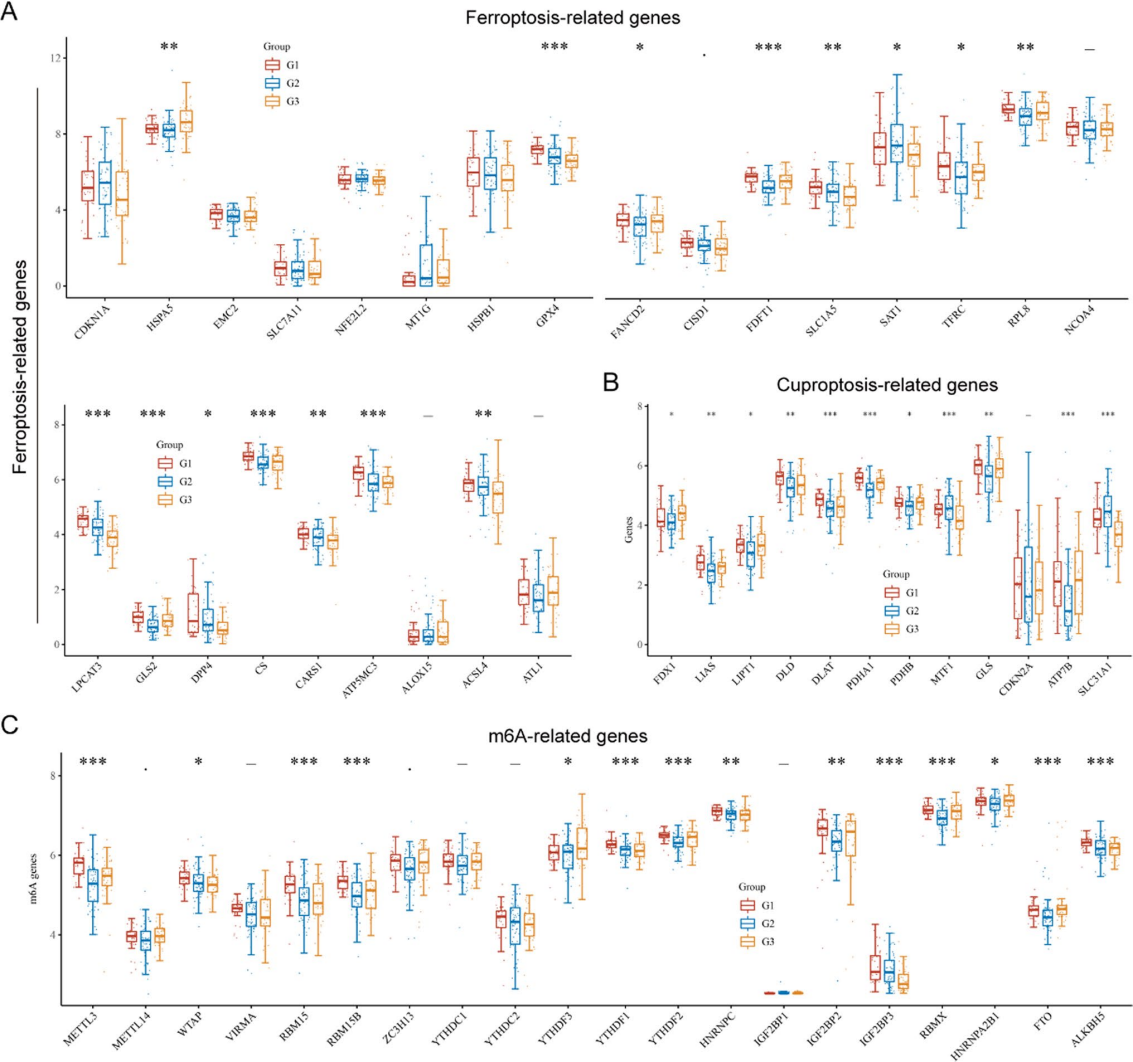
The expression of ferroptosis, cuproptosis and m6A related genes in three AML molecular subtypes. **A** Ferroptosis-related genes expression in three AML molecular subtypes. **B** Cuproptosis-related genes. **C** The m6A-related genes

Emerging studies had revealed that ferroptosis- and m6A-related genes, particularly the newly discovered cuproptosis-related genes, were critical regulators in cancer development and progression. Therefore, we next sought to investigate the relative expressions of the related genes involved in ferroptosis, cuproptosis and m6A processes in the three molecular subtypes of AML. It was found that the expressions of most ferroptosis-related genes was significantly different in the three molecular subtypes of AML (Fig. 6A). In addition, all the 9 cuproptosis-related genes had obvious difference in the three AML molecular subtypes (Fig. 6B). The m6A-related genes’ expressions in the three AML molecular subtypes were also explored, and similar results with the ferroptosis-related genes were also observed that most m6A-related genes’ expressions had markedly difference in the three AML molecular subtypes (Fig. 6C).

### The immune analysis in the three AML molecular subtypes

**Fig. 7:**
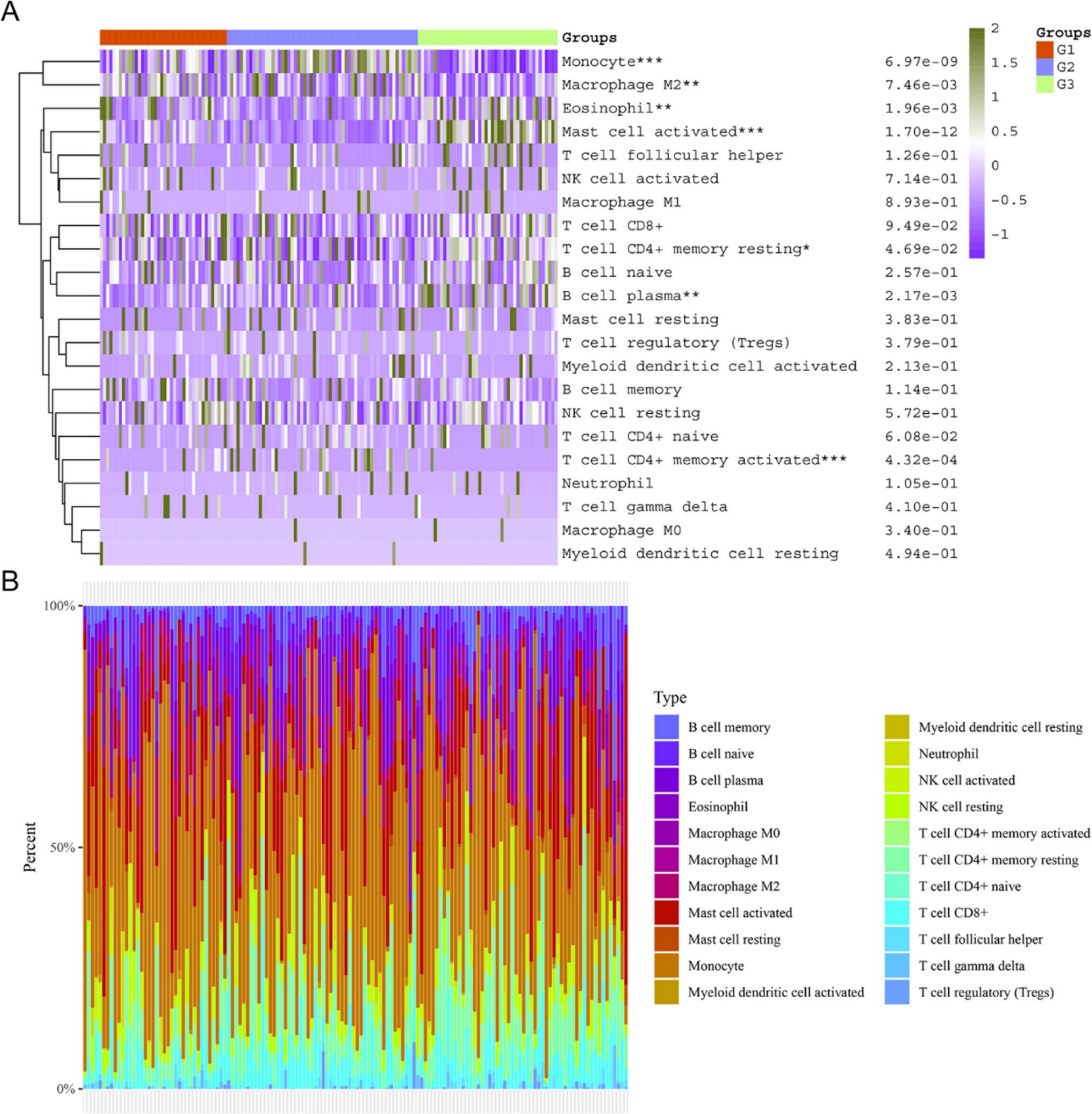
The immune scores and distribution of immune cells in three AML molecular subtypes. **A** The assays of the immune scores of diverse immune cells in three AML molecular subtypes. **B** The analysis of the distribution of immune cells in three AML molecular subtypes

**Fig. 8:**
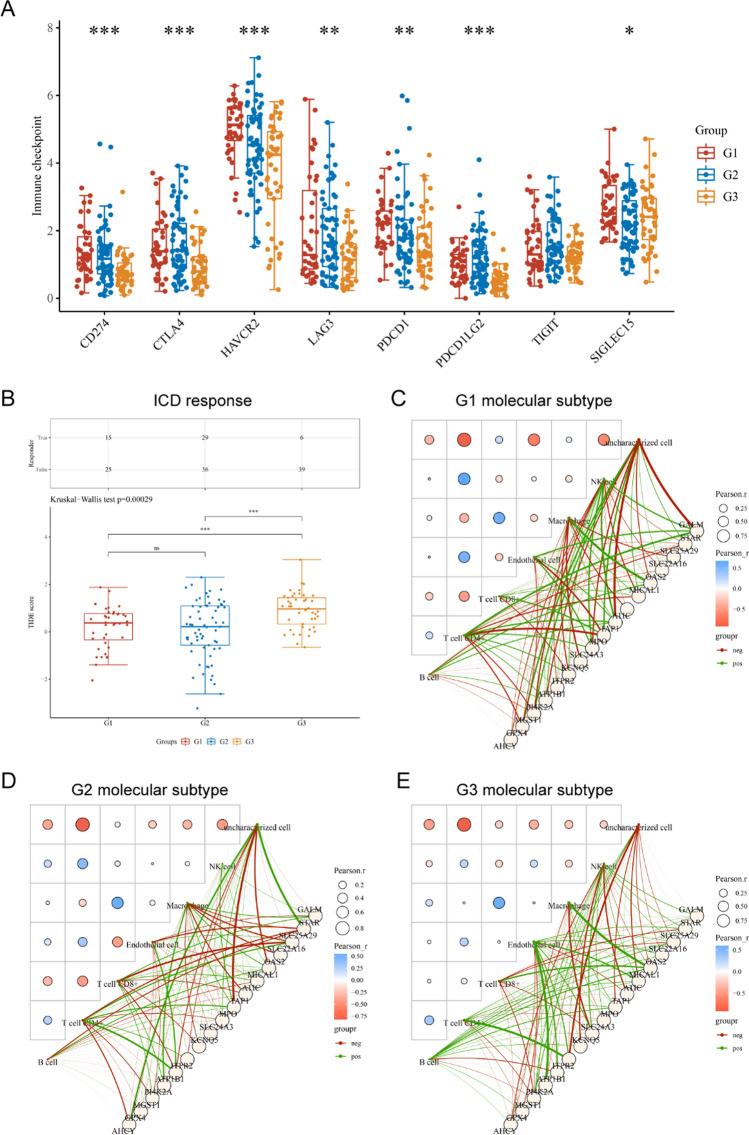
The relative expression of immune checkpoints, ICB response and the immune interacting networks construction. **A** The expressions of immune checkpoints in the three AML molecular subtypes. **B** The potential ICB response in the three AML molecular subtypes. **C**–**E** The immune interacting networks of each three AML molecular subtype-groups and the 17 overlap MRGs were also separately constructed based on EPIC algorithm. G1: Group 1; G2: Group 2; G3: Group 3

We next sought to perform the immune analysis in three AML molecular subtypes using the CIBERSORT algorithm. According to the data, we found that the immune scores of several immune cells including Monocyte, Macrophage M2, Eosinophil, T cell CD4 + memory resting, Mast cell activated and B cell plasma were remarkably different in the three AML molecular subtypes (Fig. 7A). In addition to this, we analyzed the proportion of immune cells present in each of the three molecular subtypes of AML (Fig. 7B). Besides, the relative expression of immune checkpoints in the three AML molecular subtypes were evaluated and the data suggested that nearly all the immune checkpoints including LAG3, PDCD1, PDCD1LG2, CD274, CTLA4, HAVCR2 and SIGLEC15, had remarkable difference in the three AML molecular subtypes (Fig. 8A). Then, we evaluated the potential ICB response with TIDE algorithm, our group observed that the TIDE scores were significantly higher in group 3 of AML molecular subtypes when compared with group 1 and group 2, which indicated that group 3 of AML molecular subtypes were more sensitive to ICB therapy (Fig. 8B). Additionally, the immune interacting networks of each three AML molecular subtype-groups and the 17 overlap MRGs were also separately constructed based on EPIC algorithm (Fig. 8C–E).

### Comparison of the gene and function enrichment differences in each AML molecular subtype-groups

**Fig. 9:**
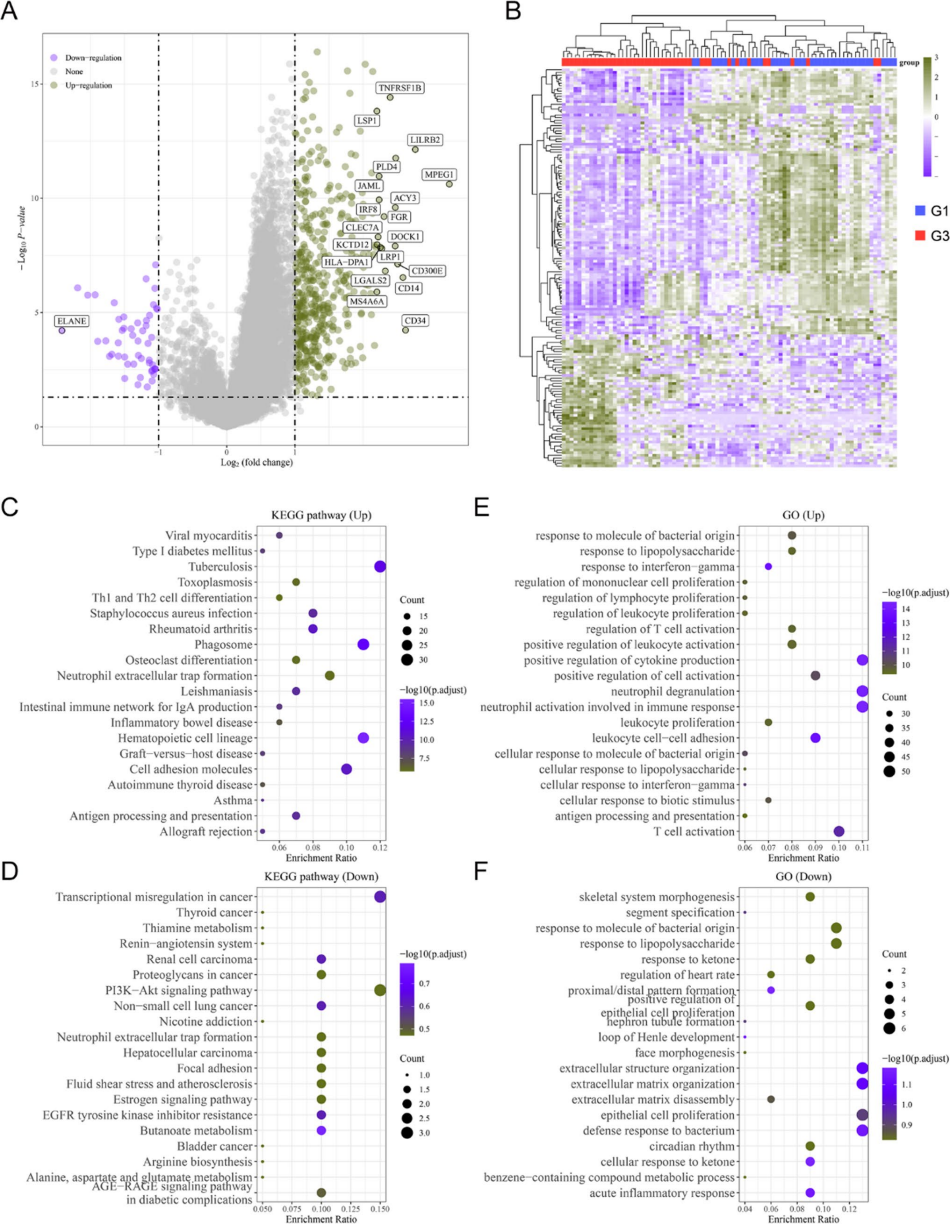
Comparison of the gene and function enrichment differences in G1 and G3 of AML molecular subtypes. **A** Volcano map. **B** Heatmap. **C **and **D** KEGG analysis. **E** and **F** GO analysis. G1: Group 1; G3: Group 3

Considering our above data indicated that there were obvious difference between group 3 (G3) and group 1 (G1) or group 2 (G2) of AML molecular subtype, we thus next attempted to investigate gene and function enrichment differences between G3 and G1 or G2. The DEGs of G1 and G3 were firstly analyzed, and 487 up-regulated genes and 50 down-regulated genes in G3 AML samples (compared with G1) were identified, and the volcano plot and heatmap were presented in Fig. 9A and B, respectively. Then, these DEGs were applied for analyzing the functional enrichment assays. The KEGG analysis indicated that the up-regulated genes were relevant with Staphylococcus aureus infection, Viral myocarditis, Type I diabetes mellitus, Tuberculosis, Toxoplasmosis and Th1 and Th2 cell differentiation, while the down-regulated genes were correlated with Renal cell carcinoma, Renin-angiotensin system, Thiamine metabolism and Thyroid cancer (Fig. 9C and D). Afterwards, the GO analysis demonstrated that the up-regulated genes were related with response to molecule of bacterial origin, regulation of mononuclear cell proliferation and regulation of lymphocyte proliferation, while the down-regulated genes were correlated with response to ketone, response to lipopolysaccharide, response to molecule of bacterial origin, segment specification and skeletal system morphogenesis (Fig. 9E and F). Afterwards, DEGs of these two groups were firstly identified, and they contained 523 up-regulated genes and 194 down-regulated genes. The comparison between G3 and G2 molecular subtypes was carried out, and both groups' DEGs were examined. The corresponding volcano map and heatmap were respectively displayed in Supplementary Figure S1A and B. The up- and down-regulated genes’ KEGG analysis were respectively presented in Supplementary Figure S1C and D, and it demonstrated that the DEGs were correlated with Systemic lupus erythematosus, Tuberculosis, Th17 cell differentiation, Type I diabetes mellitus, Viral myocarditis, Proteoglycans in cancer, Rap1 signaling pathway, Ras signaling pathway, Renin-angiotensin system and Transcriptional misregulation in cancer. In addition, the GO analysis revealed that the DEGs were relevant with regulation of T cell activation, regulation of cell–cell adhesion, response to interferon-gamma, synapse organization, xenobiotic metabolic process Supplementary Figure S1E and F).

### A prognostic model for AML was constructed based on the 17 overlap MRGs

In order to develop a risk score model for the purpose of predicting overall survival in patients with AML, the LASSO Cox regression model was applied to generate a prognostic classifier using the 17 overlap MRGs. This was done in order to fulfill the aforementioned goal. Under LASSO Cox regression analysis (Supplementary Figure S2A and B), a nine-gene signature was constructed, and the risk score formula was: Riskscore = (0.3644)*GPX4 + (− 0.2696)*ITPR2 + (− 0.0588)*SLC24A3 + (− 0.0845)*MPO + (0.1371)*ATIC + (0.0703)*OAS2 + (− 0.0367)*SLC22A16 + (− 0.0236)*STAR + (0.0768)*GALM. The samples of AML were then classified into high-risk and low-risk categories based on predetermined threshold values for risk scores. There will be more fatalities and cases of disease if the risk score is high(Supplementary Figure S2C). In addition, Patients with AML who had a high risk score had a shorter overall survival (Supplementary Figure S2D). Then, the model reliability was verified via the ROC curves analysis, and the AUC values of 1-, 3- and 5 year OS were 0.8, 0.802 and 0.81, respectively (Supplementary Figure S2E). Besides, an another method called Multivariate cox regression analysis was also utilized for obtain the prognostic model for AML based on the 17 overlap MRGs. Multivariate cox regression analysis did not reduce the gene number. Therefore, the prognostic model included 17 genes and the risk score formula was: Riskscore = (0.073)*AHCY + (0.578)*GPX4 + (− 0.0181)*MGST1 + (− 0.0374)*PI4K2A + (0.1468)*ATP1B1 + (− 0.4371)*ITPR2 + (− 0.1177)*KCNQ5 + (− 0.1157)*SLC24A3 + (− 0.0819)*MPO + (− 0.0805)*TAP1 + (0.3747)*ATIC + (− 0.1247)*MICAL1 + (0.1131)*OAS2 + (− 0.0776)*SLC22A16 + (0.0038)*SLC25A29 + (− 0.0859)*STAR + (0.2106)*GALM. After that, the training cohort was also divided into high- and low-risk groups, and the analysis of the data revealed that the high-risk group had a larger frequency of poor survival outcomes compared to the low-risk group. Additionally, a heatmap of 17 genes involved in AML was created (Supplementary Figure S2F). According to the findings of the analysis of overall survival, individuals with high risk AML had a lower overall survival time (Supplementary Figure S2G). Besides, the AUC values of 1-, 3- and 5 year OS were 0.815, 0.803 and 0.791, respectively (Supplementary Figure S2H).

## MICAL1 expression and methylation analysis in tissues and *pan*-cancers

Among these 17 overlap MRGs, we selected MICAL1 for further investigation because emerging researches indicated that MICAL1 was correlated with cancer development and progression, and its functions in AML had been not studied. Hence, we firstly investigated the MICAL1 expression in kinds of cell types from different human tissues using Harmonizome database (https://maayanlab.cloud/Harmonizome/) (Supplementary Figure S3A). Then, the expression of MICAL1 in kinds of cancer types and normal specimens were explored using TIMER 2.0 database, and the results indicated that MICAL1 expressed more highly in most tumor tissues compared with their corresponding paired normal tissues (Supplementary Figure S3B). Then, MICAL1 methylation across cancer types were investigated and the data suggested that MICAL1 methylation levels were diverse in different cancer types: MICAL1 methylation levels were high in the tumor tissues of CESC, COAD, DLBC, ESCA, LUSC, and UCEC, and MICAL1 methylation levels were low in the other cancer types’ tissue samples (Supplementary Figure S3C).

### The immune analysis of MICAL1 in *pan*-cancers

We next sought to investigate the relations between MICAL1 expression and the abundance of TILs, immunoinhibitors and chemokines in pan-cancers. It was found that MICAL1 expression was positive correlation with different TILs in most solid cancer types (Supplementary Figure S4A). Similarly, MICAL1 expression was positively relevant with nearly all the immunoinhibitors in most types of cancers except COAD, READ and SARC (Supplementary Figure S4B). In addition, the analysis of chemokines revealed that MICAL1 expression was positively relevant with most chemokines in more than half of the TCGA cancer types (Supplementary Figure S4C). Besides, since the tumor mutational burden (TMB) was closely correlated with cancer progression, we next also investigate the correlation of MICAL1 and TMB in pan-cancers, and the data suggested that MICAL1 and TMB was negatively correlated in most cancer types (Supplementary Figure S4D).

### MICAL1 is a potential prognostic factor in AML

Using the GEPIA database, we found that MICAL1 was much more expressed in AML than in GTEx normal tissues (Supplementary Figure S5A). The Sankey diagram then showed the association between MICAL1 expression and demographic variables such as age, gender, race, and survival (Supplementary Figure S5B). Subsequently, MICAL1 expression levels were used to categorize all AML samples into either a high or low group, with a higher MICAL1 expression corresponding to a higher percentage of patients who had died (Supplementary Figure S5C). In addition, according to the findings of an analysis of survival times, the overall survival of AML patients whose MICAL1 expression was high was lower than that of persons whose MICAL1 expression was low(Supplementary Figure S5D). Then, the ROC curves analysis suggested that the AUC values of 1-, 3- and 5 year OS were 0.655, 0.606 and 0.687, respectively (Supplementary Figure S5E).

### Immune filtration and immune interacting network construction of MICAL1 in AML

We next attempted to elucidate the correlations of immune infiltration and MICAL1 expression in AML. Using GSCA database, we found that MICAL1 expressions were positively relevant with Macrophage, Infiltration-Score, Exhausted infiltrate, and NKT (Supplementary Figure S6A-D), while negatively correlated with Gamma-delta infiltrate, nTreg, NK, Central-memory infiltrate, and Th17 (Supplementary Figure S6E-I). Thereafter, we constructed the immune interacting network construction of MICAL1 in AML based on CIBERSORT algorithm. The data suggested that MICAL1 was positive correlations with many immune cells such as Eosinophil, Mast cell resting, Myeloid dendritic cell activated, NK cell activated, Macrophage M0, T cell follicular helper, T cell CD4 + memory resting, T cell CD8 + , B cell plasma and B cell naive (Supplementary Figure S6J). Our suggested that MICAL1 may play an important role in regulating the activities of these immune cells.

### Building the protein interacting network of MICAL1 and functional enrichment analysis of MICAL1-related proteins

Next, the potential interacting proteins of MICAL1 was investigated by using Genemania database, and there were 20 interacting genes were found including NEDD9, SEPTIN1, INPP4B, RAB1B, RAB35, INPP4A, FLNB, CASS4, VIM, STK38, BMERB1, PLXNB2, TAS2R7, PLXNA3, EHD1, PLXNA4, PLXND1, CNPY3, PLXNC1, and COQ6 (Supplementary Figure S7A). Additionally, the correlation of each genes in AML tissues were investigated and the data suggested that most of the 20 genes were positively correlated with each other (Supplementary Figure S7B). Moreover, the functional enrichment analyses of the 20 genes were carried out. The BP of GO analysis revealed that the 20 genes were relevant with regulation of cell shape, positive regulation of axonogenesis, semaphorin-plexin pathway, regulation of cell morphogenesis, and positive regulation of cell development (Supplementary Figure S7C). Then, the CC and MF of GO analysis suggested that the 20 genes were relevant with recycling endosome membrane, cell-substrate junction, focal adhesion, endocytic vesicle, semaphorin receptor complex, semaphorin receptor activity, and phosphatase activity (Supplementary Figure S7D and E). Finally, the KEGG assays revealed that the 20 genes were enriched in inositol phosphate metabolism and axon guidance (Supplementary Figure S7F).

### Depletion of MICAL1 suppresses the proliferation of AML cells

**Fig. 10:**
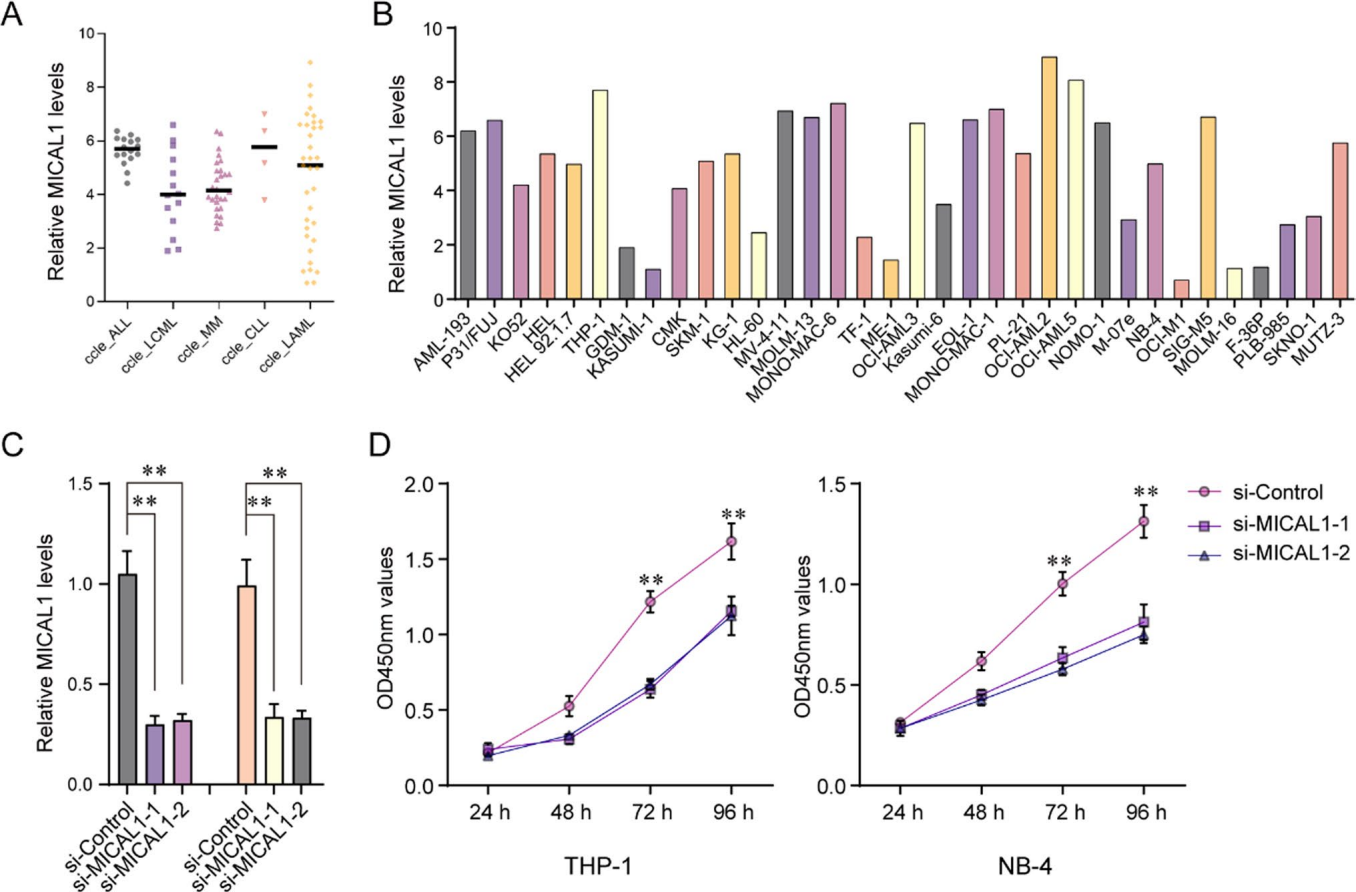
Real-time PCR assays detect MICAL1 expression and CCK-8 assays examine the cell proliferation. **A** MICAL1 mRNA levels in leukemia cell lines based on CCLE datasets. **B** MICAL1 mRNA levels in AML cell lines based on CCLE datasets. **C** The qRT-PCR assays determined the MICAL1 mRNA levels in TPH-1 and NB-4 cells after transfecting MICAL1 siRNAs. **D** CCK-8 assays. ***P < 0.001; **P < 0.01; *P < 0.05

We then downloaded the mRNA expression data of the leukemia cell lines including ALL, CML, MM (multiple myeloma), chronic lymphoid leukemia), AML to investigate the mRNA expression levels of MICAL1 from CCLE database. The data suggested that MICAL1 expressed more highly in ALL, CLL and AML than that in CML and MM, though AML expressed diverse MICAL1 mRNA levels (Fig. 10A). Then, the MICAL1 mRNA levels in 34 AML cell lines were also investigated, and it was found that THP-1, OCI-AML2 and OCI-AML5 expressed the most highly MICAL1 mRNA levels (Fig. 10B). Next, The functions of MICAL1 in classical AML cell lines were something we wanted to investigate. We generated the siRNAs targeting MICAL1 in order to accomplish this goal. These siRNAs, together with control siRNAs, were respectively transfected into AML cell lines TPH-1 and NB-4 cells. The results of qRT-PCR assays indicated that the MICAL1 siRNAs could obviously reduce the levels of MICAL1 in both TPH-1 and NB-4 cells (Fig. 10C). Thereafter, the CCK-8 assays were utilized for detecting the affection of MICAL1 siRNAs transfection on the cellular growth of TPH-1 and NB-4 cells, and the results demonstrated that decreased MICAL1 remarkably suppressed the AML cells proliferation (Fig. 10D).

### MICAL1 is correlated with multiple *cancer*-related pathways

**Fig. 11:**
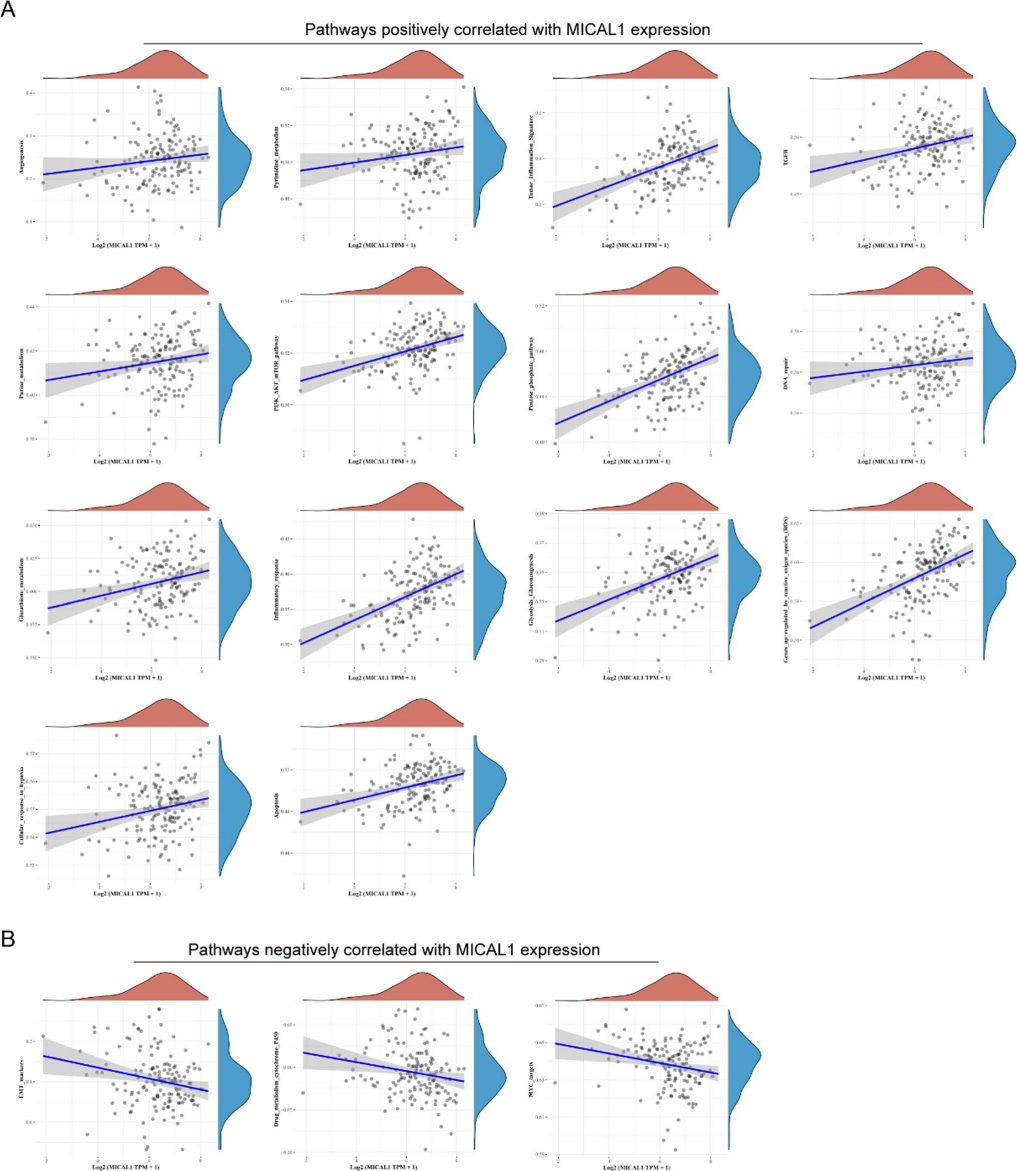
MICAL1 was related with kinds of the cancer-related pathways. **A** Pathways positively correlated with MICAL1 expressions. **B** Pathways negatively correlated with MICAL1 expressions

Next, we attempted to investigate the relationships between MICAL1 and kinds of cancer-related pathways including cellular response to hypoxia, tumor inflammation signature, MYC targets, Purine-metabolism, Drug-metabolism-cytochrome-P450, DNA replication, Pentose-phosphate-pathway, G2M checkpoint, P53 pathway, Glutathione-metabolism, angiogenesis, collagen formation, degradation of ECM, DNA repair, genes up-regulated by ROS, IFNG signaling, PI3K/AKT/mTOR pathway, EMT markers, Pyrimidine-metabolism, apoptosis, TGFB signaling, Inflammatory-response, hallmark glycolysis, hypoxia signature and metabolism related pathways. The R software “GSVA” package was used to analyze, choosing parameter as method = “ssgsea”. Spearman's correlation coefficient was used to investigate the relationship between MICAL1 and pathway scores. The results suggested that MICAL1 was significantly positively correlated with angiogenesis, Pyrimidine-metabolism, tumor inflammation signature, TGFB, Purine_metabolism, PI3K/AKT/mTOR pathway, Pentose-phosphate-pathway, DNA repair, Glutathione-metabolism, Inflammatory-response, etc. (Fig. 11A). And MICAL1 was significantly negatively correlated with EMT markers, Drug-metabolism-cytochrome-P450, and MYC targets (Fig. 11B).

## Discussion

Recently, there have been significant advancements in the research of diagnostic and prognostic markers for AML. These developments have contributed to a deeper understanding and improved management of this disease [27]. The discovery of molecular genetic markers has provided new tools for the diagnosis and prognostic assessment of AML. Markers such as FLT3, NPM1, CEBPA, IDH1, and IDH2 have been identified [14, 28]. These markers not only assist in confirming the diagnosis of AML but also have the capability to predict the disease prognosis, offering crucial guidance for treatment decisions. RNA sequencing have been widely employed in AML research. These technologies aid in distinguishing various AML subtypes, predicting disease progression and survival rates in patients, and providing valuable information for personalized treatment [29–31]. On the other hand, an increasing amount of research is concentrating on cellular and molecular markers within the AML microenvironment. These markers help elucidate the interactions between AML and the immune system, hematopoietic cells, and other components of the microenvironment, offering insights for developing novel immunotherapy strategies [32, 33]. However, more sensitive prognostic markers are of paramount importance for AML patients. These markers can provide more accurate disease prognosis information, aiding both physicians and patients in better comprehending the disease's progression and treatment outlook.

AML patients’ leukemia cells typically exhibit abnormal metabolic features. These include aberrant energy metabolism, often characterized by a high reliance on the glycolytic pathway, a phenomenon known as the “Warburg effect.” The metabolic abnormalities in AML cells also manifest as disruptions in lipid metabolism, amino acid metabolism, and nucleotide metabolism [34–36]. There is a mutual association between metabolic dysregulation and the pathogenesis of AML. Disruptions in certain metabolic pathways can lead to abnormal proliferation and differentiation obstacles in hematopoietic stem cells of AML patients, ultimately resulting in the excessive proliferation and accumulation of leukemia cells. In this study, we employed multiple data sources and analytical tools to systematically screen and identify differentially expressed genes in AML. As a result, we successfully identified 17 potential prognostic markers. Then, we performed pan-cancer assays and the 17 overlapping MRGs exhibit varying expression patterns in different cancer types and may play crucial roles in the clinical progression and survival of cancer. Importantly, the majority of the 17 MRGs have a significant impact on OS or PFS in multiple cancer types, suggesting that these genes play crucial roles in the development and treatment of cancer, and their abnormal expression may be associated with cancer progression or treatment response. These MRGs may hold potential clinical value and could serve as significant factors for cancer patient prognosis assessment and treatment decisions.

GSVA is a method used to analyze gene expression data, aiming to assess the activity or enrichment level of gene sets (typically associated with biological functions, pathways, or phenotypes) between different samples [37, 38]. The primary objective of GSVA is to transform gene expression data into enrichment scores for gene sets, enabling a more comprehensive understanding of the activity level of gene sets across different samples or conditions [39]. We found that the 17 overlapping metabolic-related genes (MRGs) exhibit differential GSVA scores across various cancer types. Typically, these MRGs demonstrate higher GSVA scores in tumor samples compared to their corresponding normal tissues, suggesting potential abnormal activity or enrichment in the majority of cancers. Additionally, the study reveals associations between GSVA scores and clinical stages as well as pathological grading, implying a correlation between the activity of these MRGs and cancer progression and grading. Further analysis uncovers correlations between GSVA scores and the activity of multiple signaling pathways. GSVA scores positively correlate with apoptosis, EMT, and ER signaling pathways, while negatively correlating with cell cycle and DNA damage pathways in most cancer types. These findings underscore the potential roles and relevance of these 17 MRGs across multiple cancer types, which may facilitate further investigation into their biological functions and clinical significance.

The mechanism behind AML’s development is highly complex, involving multiple genetic factors and biological variables. Therefore, to better comprehend this complexity, researchers may choose to simultaneously consider multiple genes to conduct a more comprehensive analysis of the interactions among different factors. Using multiple genes for classification can enhance the accuracy of the classification. A single gene might not provide sufficient information to classify patients accurately, while a combination of multiple genes can better reflect the complexity of the disease. Then, we used 17 MRGs to divide the 150 AML samples into these 3 molecular subtypes. Ferroptosis, cuproptosis, and m6A are biological processes related to cell death and gene regulation, and they are associated with the occurrence and development of leukemia. Specifically, ferroptosis is a form of cell death that may be dysregulated in certain types of leukemia, particularly AML. Iron ion metabolism and lipid peroxidation may be linked to the survival and proliferation of AML cells. On the other hand, cuproptosis is a cell death process mediated by copper ions, and although its association with leukemia is not yet fully explored, recent research suggests that genes related to copper ion metabolism may play a role in leukemia [40, 41]. Additionally, m6A modification is a methylation modification on RNA that is crucial for RNA stability and translation regulation. In some types of leukemia, m6A modification may be dysregulated, potentially affecting the development and proliferation of leukemia cells [42, 43]. Then, we investigate the relative expression of genes related to ferroptosis, cuproptosis, and m6A processes in the three molecular subtypes of AML. Importantly, we observed that the expression of ferroptosis-related genes, cuproptosis-related genes and m6A-related genes exhibited a dysregulated level in in the three AML molecular subtypes based on 17 MRGs. Our finding suggested that the expression patterns of these 17 genes in different AML subtypes are influenced by these cellular death and gene regulation processes, or they may play a certain role in regulating these processes. Othe other hand, we found diversity in the immune characteristics among different AML molecular subtypes, including variations in the composition of immune cells and the expression of immune checkpoints. This is significant for understanding the immune status of AML patients and potential treatment strategy considerations. Particularly, the third group of AML molecular subtypes appears to be more sensitive to immune checkpoint blockade therapy, providing valuable insights for personalized treatment approaches.

Multi-gene prognostic models have several advantages compared to single-gene prognostic models. Firstly, multi-gene models can provide more comprehensive information as they consider the expression of multiple genes, which better reflects the complexity and diversity of diseases. Secondly, multi-gene models are often more accurate because they rely on combinations of multiple genes for prediction, reducing the chance of random errors in predictions. Additionally, multi-gene models can take into account the interactions between genes, aiding in a deeper understanding of how gene networks influence diseases. Most importantly, multi-gene models provide a stronger basis for personalized treatment, allowing for the developments of more precise treatment strategies based on the expressions of multiple genes, thereby improving treatment outcomes. Previously, several studies have developed diagnostic models based on MRGs in several types of tumors, such as lung cancer, cervical cancer and rectal cancer. However, the prognostic model based on MRGs in AML was rarely reported. Then, we performed LASSO using the 17 MRGs and developed a now prognostic model using GPX4, ITPR2, SLC24A3, MPO, ATIC, OAS2, SLC22A16, STAR and GALM. Patients diagnosed with AML who had a high risk score had a poorer overall survival rate, according to survival tests. This was also suggested by the overall survival analysis. The possibility of the new model to be employed as an innovative prognostic model for patients with AML was brought to light by our findings.

Among the 17 MRGs, our attention focused on MICAL1. MICAL1 belongs to the MICAL (Molecule Interacting with CasL) protein family. It contains multiple domains, with the most significant being the C-terminal domain of MICAL protein [44]. This domain possesses monooxygenase activity, allowing it to oxidize hydrophilic amino acid tyrosine on actin filaments. This oxidation process, in turn, regulates the dynamic changes in the cell cytoskeleton. The monooxygenase activity of MICAL1 makes it a crucial regulatory factor in intracellular oxidative stress responses. Under certain cellular stress conditions [45, 46], MICAL1 can promote the depolymerization of actin filaments, leading to changes in the cell cytoskeleton. It is essential for the cell's response to oxidative stress. Several studies have reported that MICAL1 was involved in the progression of several tumors. For instance, Cai et al. reported that knockdown of MICAL1 inhibited pancreatic cancer cell proliferation, migration, and invasion by activating the WNT/-catenin pathway, which was significantly expressed in pancreatic cancer [47]. Deng et al. showed that in breast cancer, MICAL1 was overexpressed, and its effect on proliferation was seen through the maintenance of cyclin D expression via ROS-sensitive PI3K/Akt/ERK signaling [48]. However, expression of MICAL1 in AML as well as its function are not known due to a lack of research. In this study, we found that MICAL1 expression was distinctly increased in many types of tumors, including AML, suggesting it as a tumor promotor. In addition, we confirmed that high MICAL1 expression predicted a poor clinical prognosis. Moreover, our results suggest that MICAL1 may have a multifaceted function in immunological regulation in AML, with positive correlations to the infiltration and activity of some immune cell types and negative correlations to other immune cell types. Moreover, we found that MICAL1 expression is positively correlated with macrophage infiltration, infiltration score, exhausted infiltrate, and natural killer T cells (NKT) in AML, suggesting that MICAL1 may influence AML progression by modulating the behavior and activity of these cells. Conversely, MICAL1 expression is negatively correlated with γδ T cells, natural regulatory T cells (nTreg), natural killer cells (NK), central memory T cells, and Th17 cell infiltration, which typically possess anti-tumor functions. Elevated MICAL1 expression may suppress the infiltration or function of these cells, thereby promoting AML development. Overall, high expression of MICAL1 in AML may affect the infiltration and function of various immune cells, contributing to disease progression. Future studies should further explore the specific molecular mechanisms of MICAL1 in these processes to better understand its role in AML and evaluate its potential as a therapeutic target. Finally, we concluded our functional tests by showing that MICAL1 knockdown markedly reduced AML cell proliferation, providing further evidence for its role as a tumor promotor in AML.

## Conclusions

We identified 17 deferentially expressed survival-related MRGs in AML and clarified the potential molecular subtypes of AML based on these 17 overlap MRGs. Then, we developed a prognostic model using 9 MRGs, which suggested a strong prognostic value for AML patients. In addition, we analyzed the association between 17 overlap MRGs and TME. Finally, we carried out functional investigations and established that MICAL1 was substantially expressed in AML cells. We also found that knocking down MICAL1 significantly inhibited the growth of AML cells. Our study has provided valuable insights into the molecular subtyping, prognosis assessment, and potential therapeutic strategies for AML. It offers new hope for improving the survival and quality of life for AML patients. Future research will continue to delve into these directions, aiming to provide further scientific foundations for precision medicine and treatment of AML.

### Supplementary Information

Supplementary Materials 1.

## Data Availability

The datasets generated during and/or analyzed during the current study are available from the corresponding author upon reasonable request.
