# Supplementary material for: MICAL1 promotes the proliferation in acute myeloid leukemia and is associated with clinical prognosis and immune infiltration
Source: Discov Oncol. 2024 Jul 12;15:279. doi: 10.1007/s12672-024-01150-6 (PMC11245461; doi:10.1007/s12672-024-01150-6)
Supplement: Supplementary file 1 — Supplementary Materials 1. [file 12672_2024_1150_MOESM1_ESM.docx]

**Supplementary materials**


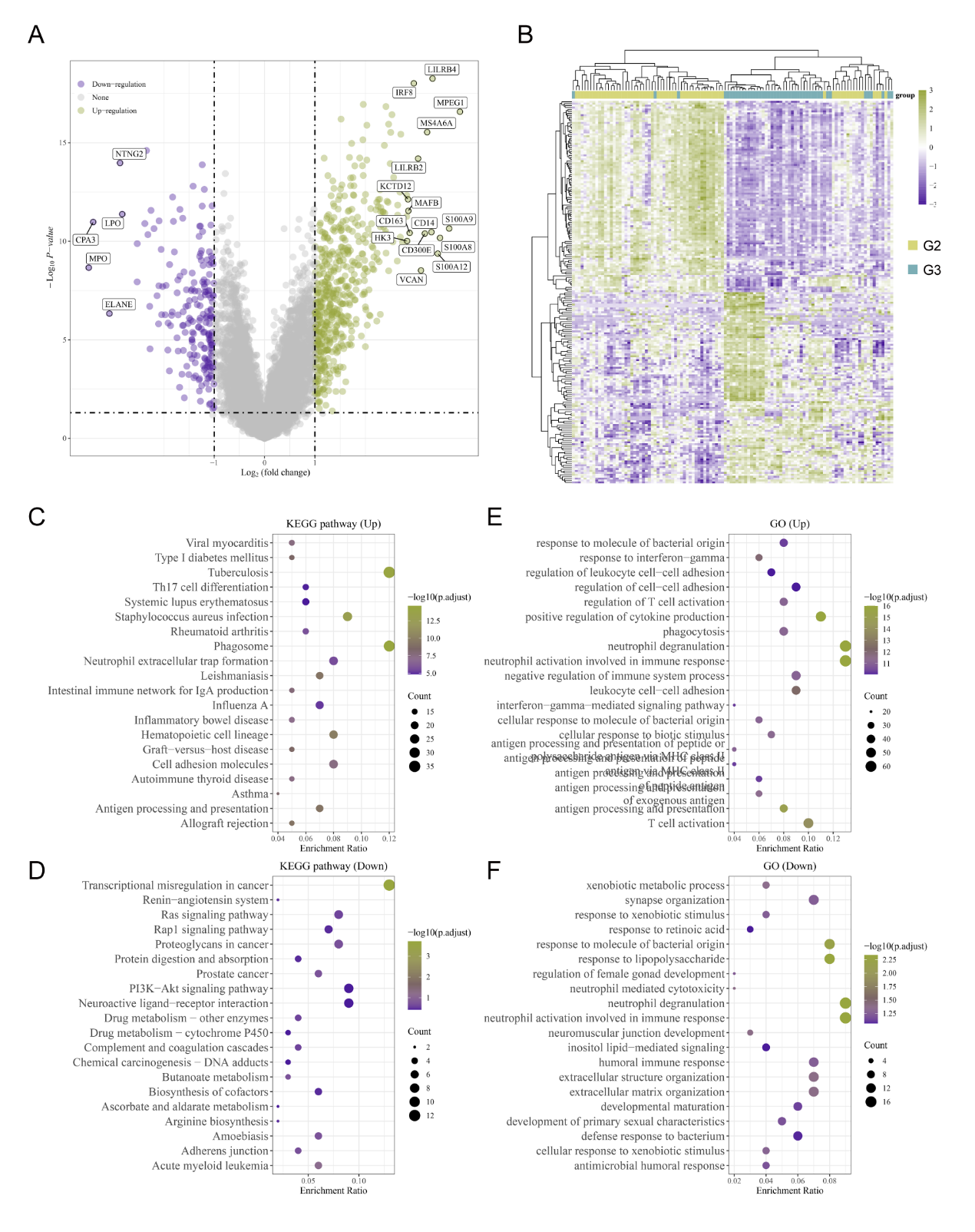


**Supplementary Figure S1. Comparison of the gene and function enrichment differences in G2 and G3 of AML molecular subtypes.** (A) Volcano map. (B) Heatmap. (C and D) KEGG analysis. (E and F) GO analysis. G2: Group 2; G3: Group 3.


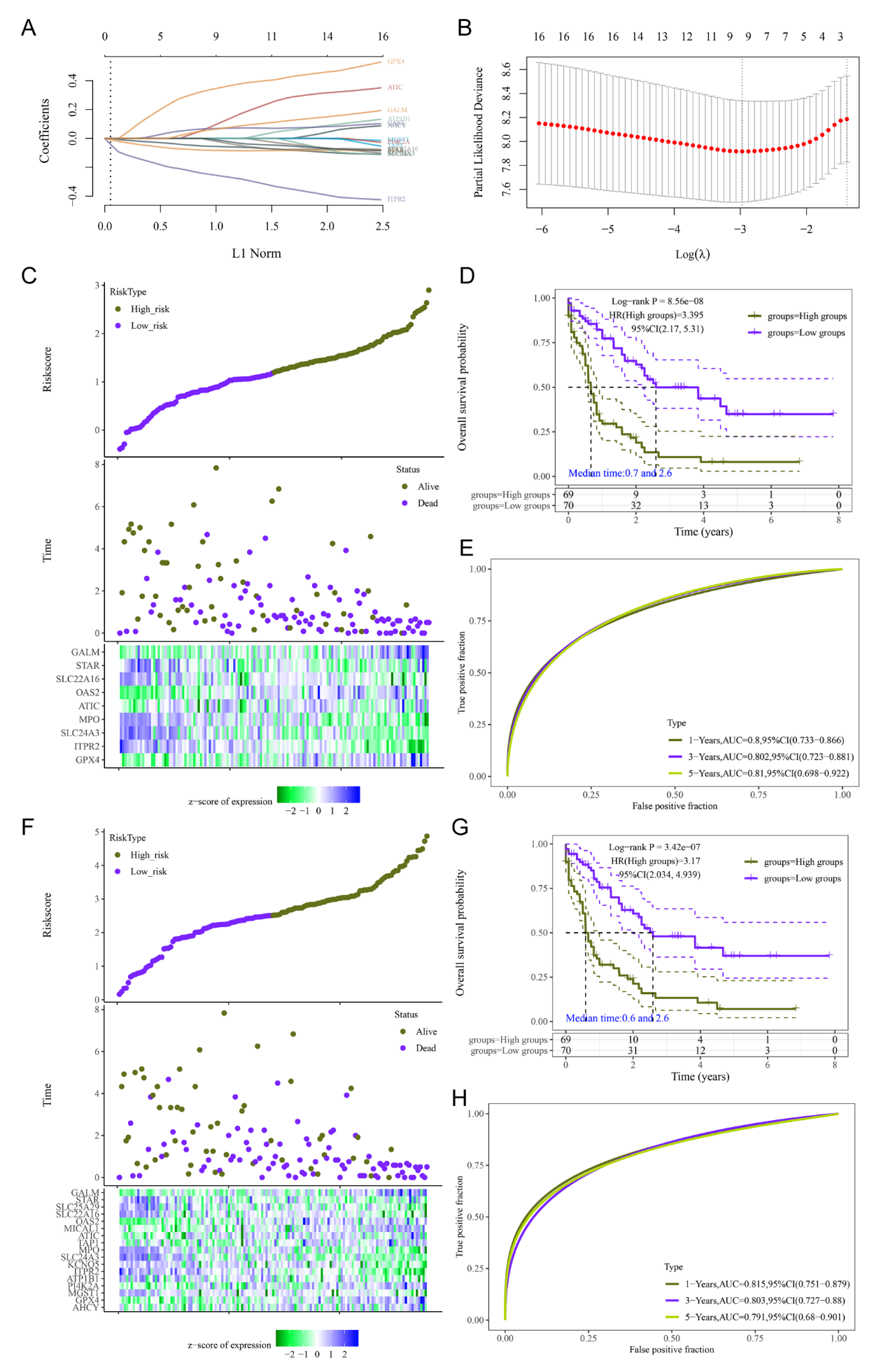


**Supplementary Figure S2. A potential prognostic model for AML based on the 17 overlap MRGs.** (A) Plots of the LASSO coefficients. LASSO, by adding an L1 regularization term (absolute value penalty) to the linear regression loss function, tends to shrink the coefficients of certain features, even to the point of making them zero. This means that LASSO can automatically select the most important features, helping to improve the model's generalization ability and reduce the risk of overfitting. (B) Using cross-validation to pick LASSO Cox model parameters. (C) Nine genes' expression patterns in high- and low-risk groups, ranked from highest to lowest according to risk. (D) Overall survival analysis of high and low risk groups. (E) Time-dependent ROC curves for 1-, 3- and 5-year OS. (F) The relative expression levels of 17 genes in high- and low-risk groups, as well as the risk score distribution (up and down) (G) Overall survival analysis of high and low risk groups. (H) Time-dependent ROC curves for 1-, 3- and 5-year OS.


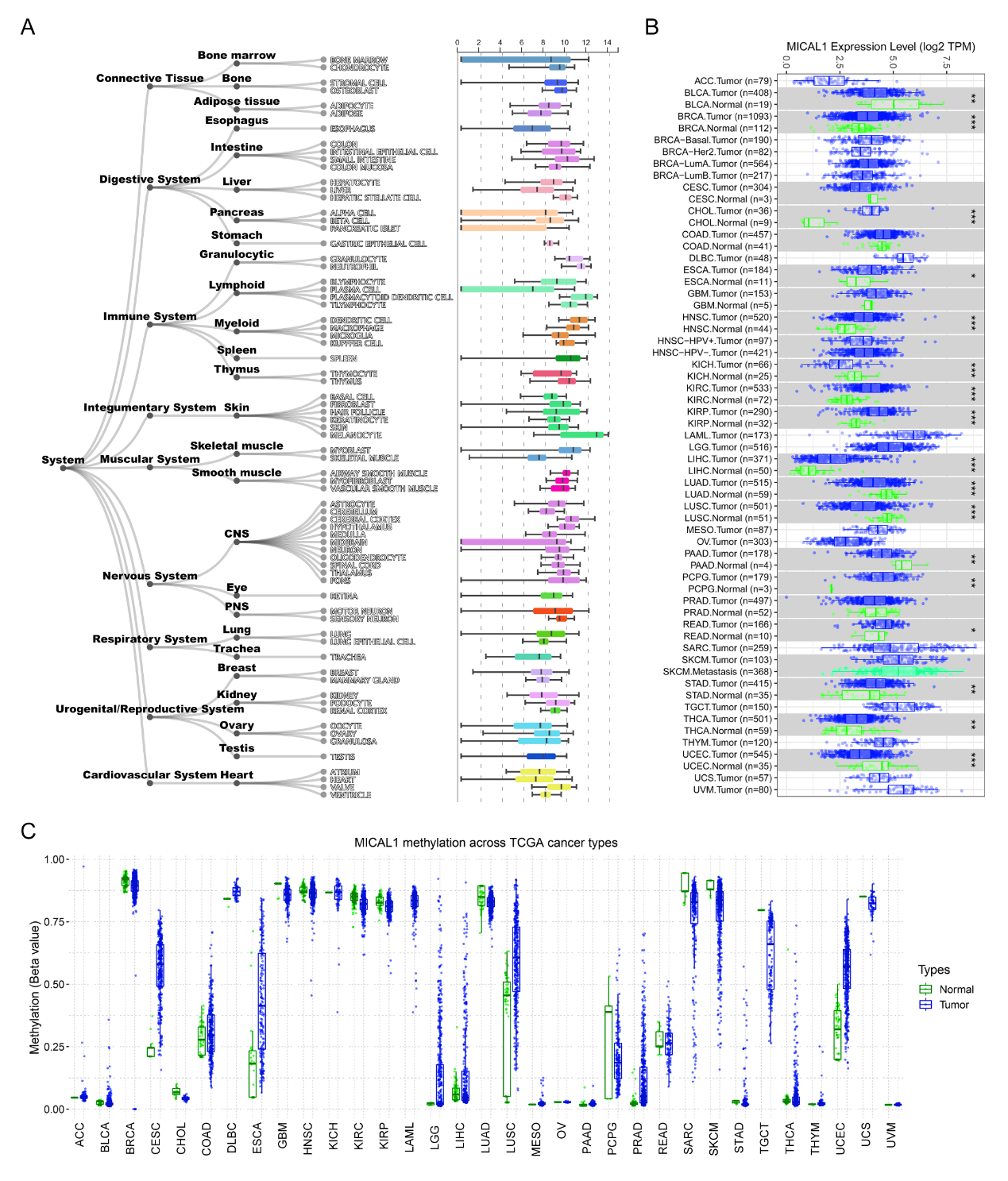


**Supplementary Figure S3. MICAL1 expression and methylation analysis.** (A) The MICAL1 expression in kinds of cell types from different human tissues. (B) The expression of MICAL1 in kinds of cancer types and their corresponding paired normal tissues. (C) MICAL1 methylation across cancer types.


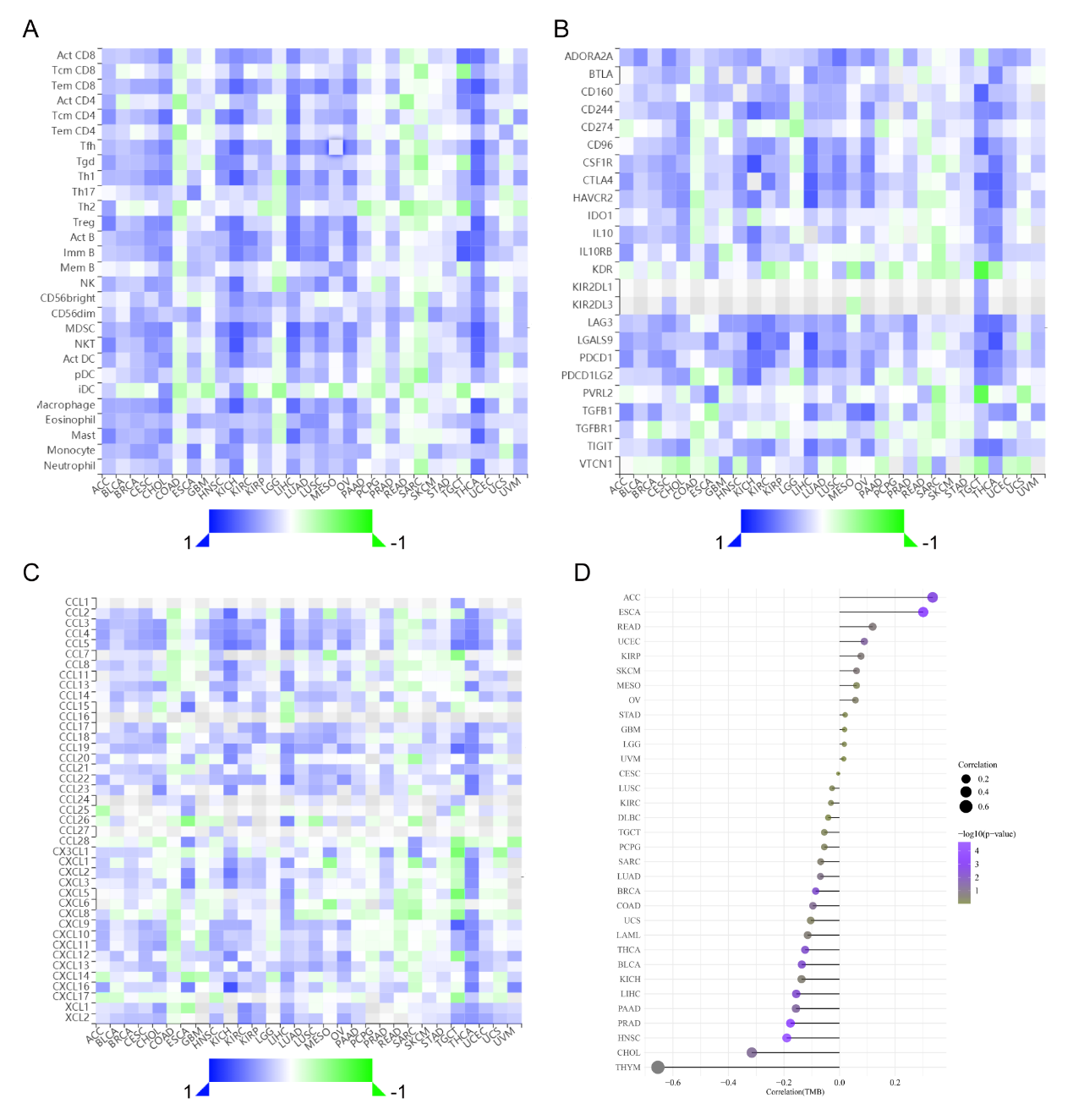


**Supplementary Figure S4. MICAL1 immune analysis in cancers.** (A) The relations between MICAL1 expression and the abundance of tumor-infiltrating lymphocytes (TILs) in cancers. (B) The relations between MICAL1 expression and the immunoinhibitors in pan-cancers. (C) The relations between MICAL1 expression and the chemokines in pan-cancers. (D) The correlation of MICAL1 and tumor mutational burden (TMB) in pan-cancers.


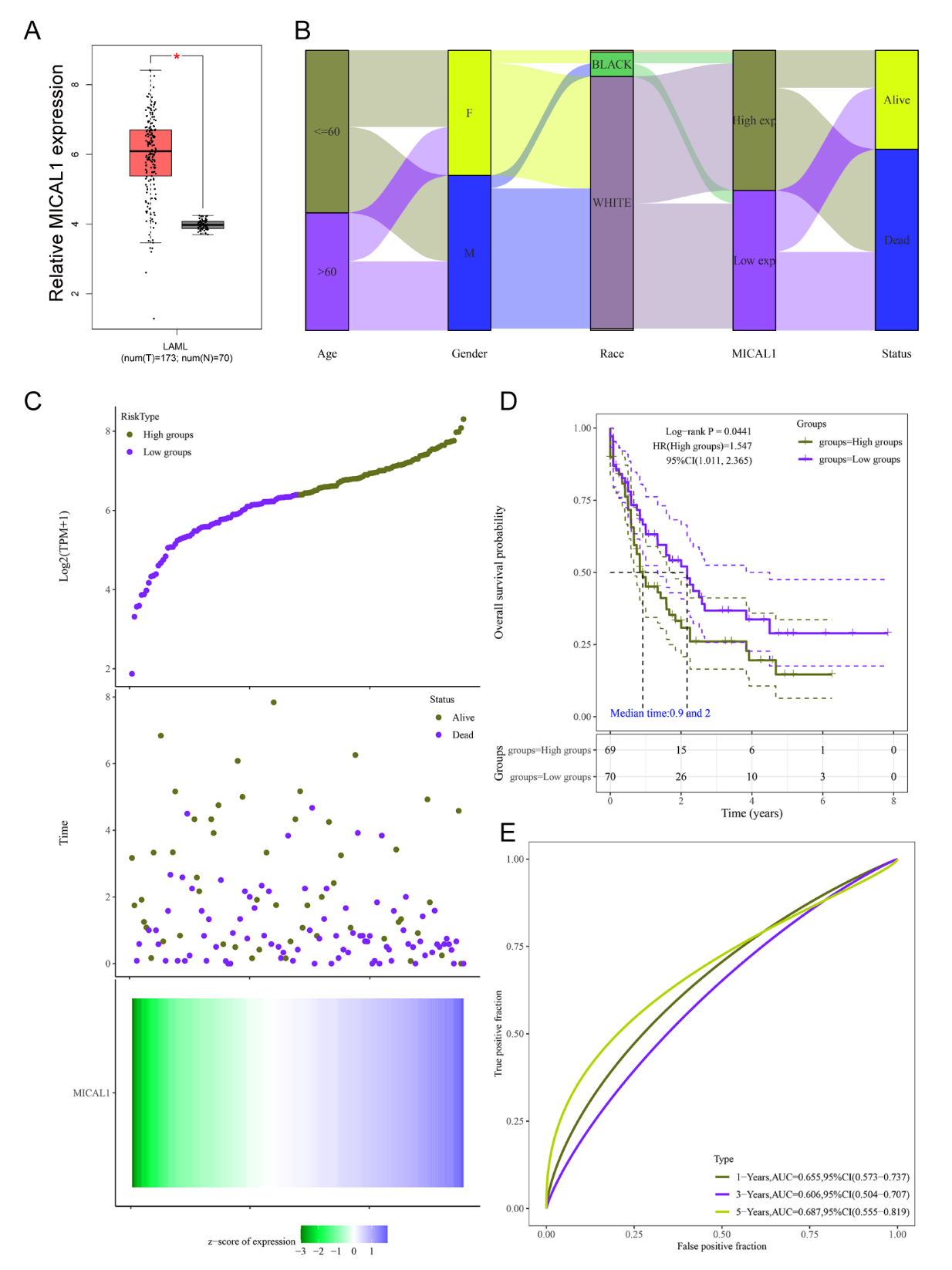


**Supplementary Figure S5. MICAL1 might be used as a potential prognostic factor in AML.** (A) MICAL1 relative expression in AML samples from GEPIA database. (B) The Sankey diagram displayed the correlation of MICAL1 expression and patients’ clinical features. (C) The risk score rank (up, based on MICAL1 expression levels), distribution of survival status (alive or dead; middle) and expression patterns of MICAL1 in high- and low-risk groups. (D) Overall survival analysis. (E) Time-dependent ROC curves for 1-, 3- and 5-year OS.


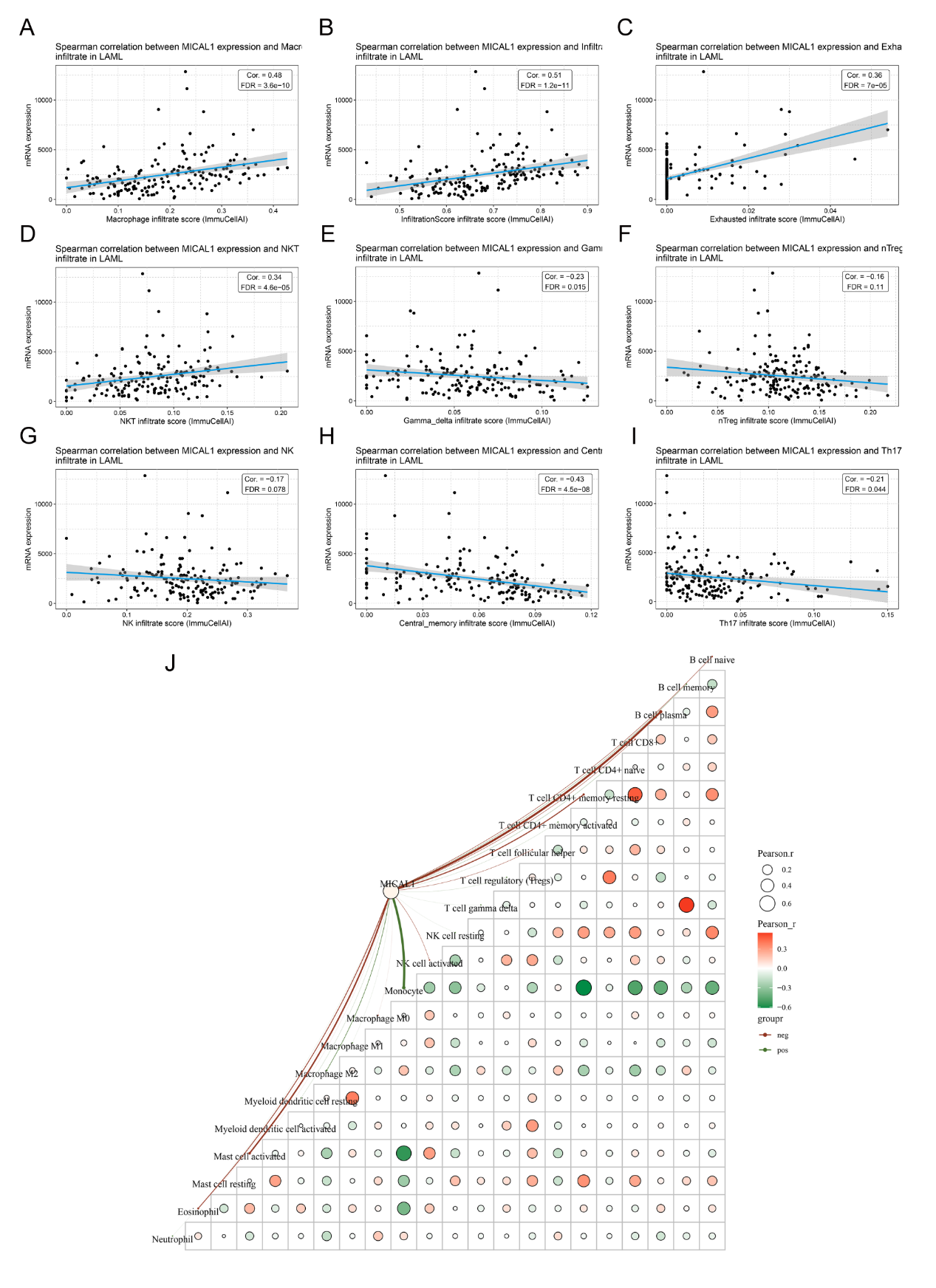


**Supplementary Figure S6. The correlation of immune infiltration and MICAL1 expression in AML.** The correlations between MICAL1 expressions and Macrophage (A), Infiltration-Score (B), Exhausted infiltrate (C), and NKT (D), Gamma-delta infiltrate (E), nTreg (F), NK (G), Central-memory infiltrate (H), and Th17 (I). (J) Constructing the immune interacting network of MICAL1 in AML.


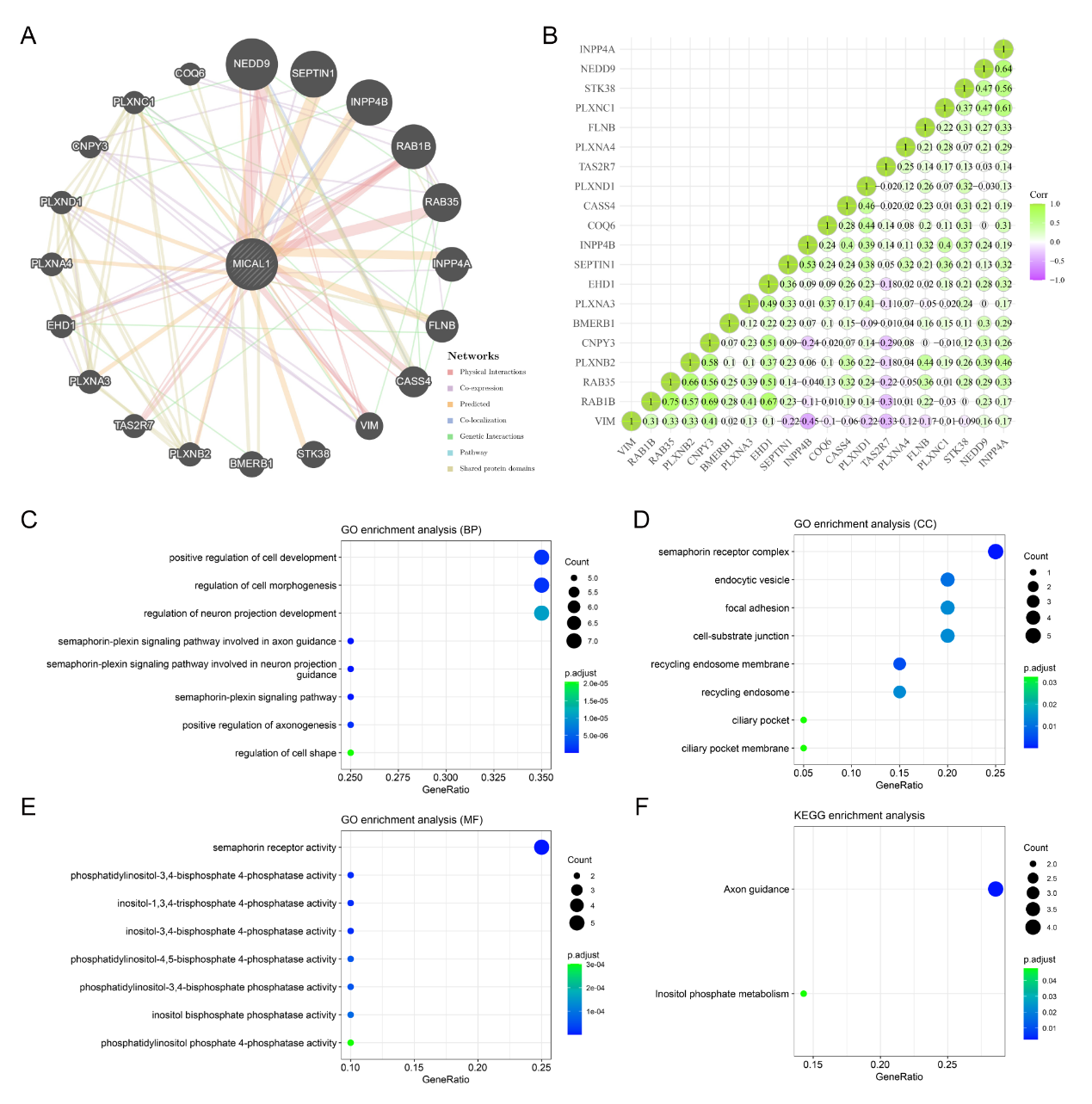


**Supplementary Figure S7. The interacting network of MICAL1.** (A) The potential interacting network of MICAL1 was constructed by using Genemania database. (B) The correlation of each MICAL1-interacting genes in AML tissues. (C-E) Gene ontology (GO) analysis of the 20 MICAL1-interacting genes. (F) KEGG analysis of the 20 MICAL1-interacting genes.
